# DNA methylation and smoking in Korean adults: epigenome-wide association study

**DOI:** 10.1186/s13148-016-0266-6

**Published:** 2016-09-22

**Authors:** Mi Kyeong Lee, Yoonki Hong, Sun-Young Kim, Stephanie J. London, Woo Jin Kim

**Affiliations:** 1 Epidemiology Branch, National Institute of Environmental Health Sciences, Department of Health and Human Services, National Institutes of Health, Research Triangle Park, Durham, NC 27709 USA; 2 Institute of Medical Science, Kangwon National University, Chuncheon-si, Gangwon-do 24341 South Korea; 3 Department of Internal Medicine and Environmental Health Center, Kangwon National University Hospital, School of Medicine, Kangwon National University, Chuncheon-si, Gangwon-do 19300 South Korea; 4 Institute of Health and Environment, Seoul National University, Seoul, 08826 South Korea

**Keywords:** DNA methylation, Smoking, Epigenome-wide association study, Cotinine, Duration of smoking cessation, Gene expression

## Abstract

**Background:**

Exposure to cigarette smoking can increase the risk of cancers and cardiovascular and pulmonary diseases. However, the underlying mechanisms of how smoking contributes to disease risks are not completely understood. Epigenome-wide association studies (EWASs), mostly in non-Asian populations, have been conducted to identify smoking-associated methylation alterations at individual probes. There are few data on regional methylation changes in relation to smoking. Few data link differential methylation in blood to differential gene expression in lung tissue.

**Results:**

We identified 108 significant (false discovery rate (FDR) < 0.05) differentially methylated probes (DMPs) and 87 significant differentially methylated regions (DMRs) (multiple-testing corrected *p* < 0.01) in current compared to never smokers from our EWAS of cotinine-validated smoking in blood DNA from a Korean chronic obstructive pulmonary disease cohort (*n* = 100 including 31 current, 30 former, and 39 never smokers) using Illumina HumanMethylation450 BeadChip. Of the 108 DMPs (FDR < 0.05), nine CpGs were statistically significant based on Bonferroni correction and 93 were novel including five that mapped to loci previously associated with smoking. Of the 87 DMRs, 66 were mapped to novel loci. Methylation correlated with urine cotinine levels in current smokers at six DMPs, with pack-years in current smokers at six DMPs, and with duration of smoking cessation in former smokers at eight DMPs. Of the 143 genes to which our significant DMPs or DMRs annotated, gene expression levels at 20 genes were associated with pack-years in lung tissue transcriptome data of smokers (Asan Biobank, *n* = 188).

**Conclusions:**

Our study of differential methylation in Koreans confirmed previous findings from non-Asian populations and revealed novel loci in relation to smoking. Smoking-related differential methylation in blood is associated with gene expression in lung tissue, an important target of adverse health effects of smoking, supporting the potential functional importance of methylation in smoking-related disease.

**Electronic supplementary material:**

The online version of this article (doi:10.1186/s13148-016-0266-6) contains supplementary material, which is available to authorized users.

## Background

Smoking is well-known for its adverse health effects [1]; however, between 10 and 35 % of people still smoke daily worldwide [2]. Despite established evidence of the causal relationships between smoking and elevated risk of diseases including cancers [3] and pulmonary [4] and cardiovascular diseases [5], the underlying mechanisms are not completely understood. One proposed mechanism is through DNA methylation.

DNA methylation, a type of epigenetic modification, plays a key role in regulating gene expression [6]. Unlike DNA sequence, methylation has cell-type and tissue-specific characteristics. DNA methylation can be impacted by age [7], gender [8], and exposures such as obesity [9] and smoking [10].

At least 16 epigenome-wide association studies (EWASs) of the association between smoking and blood DNA methylation in adults have been published [11–26]. Only one study was conducted in an East Asian population [26]; most have been conducted in populations of European ancestry with others in African American, Arab, and South Asian populations. There is no study in Koreans. There are few data where reported smoking has been biochemically validated [11, 21, 25] or where methylation has been evaluated in relation to quantitative biomarkers of smoking [21, 27], pack-years, or duration of smoking cessation [22–25]. Only one EWAS correlated differential methylation in blood with gene expression in lung tissue, and only one locus was examined in 10 individuals [19].

The published EWASs of smoking have identified individual differentially methylated probes (DMPs) rather than differentially methylated regions (DMRs). Identification of DMRs associated with an exposure can provide stronger evidence for causality than single DMPs [28]. In addition, DMR analysis is statistically more powerful for detection of association with disease traits or exposures [29].

To identify both DMPs and DMRs in relation to smoking, we conducted an EWAS in 100 adults from a Korean chronic obstructive pulmonary disease (COPD) cohort using the Infinium HumanMethylation450 BeadChip (450k). For the DMPs of genome-wide significance, we investigated their relationship with smoking intensity (urine cotinine) and cumulative smoking (pack-years) in current smokers and duration of smoking cessation in former smokers. As a replication look-up, we also evaluated association between methylation and smoking at previously published probes in our data. For the loci to which significant DMPs or DMRs mapped, we examined differential transcriptome profiles in relation to pack-years in lung tissue from a separate population—188 smokers from the Asan Biobank [30].

## Methods

### Study participants and exposure to cigarette smoking: the Korean COPD cohort

We aimed to compare methylation in current and former smokers separately to never smokers. For this purpose, we measured DNA methylation in 100 of 190 participants in a Korean COPD cohort [31]. Of the 100 participants, 60 had COPD and 40 were without COPD. The breakdown by smoking was 39 never, 30 former, and 31 current smokers. Subjects were recruited from a rural area in Korea. Having available clinical information, computed tomography (CT) data, survey questionnaire, and blood/urine samples were used for sample selections of methylation profiling. Additional approximate frequency matching on age and smoking status was applied. Details of the COPD cohort have been published [31]. All study participants completed a questionnaire and provided both blood and urine samples. Urine samples were collected at the time of participants’ baseline visits. Fresh morning urine samples were obtained from subjects at the time similar with blood sampling. Urine samples had been frozen at −70 °C. Height (cm) and weight (kg) were measured twice for each participant using a body composition analyzer IOI 353 (Aarna Systems., Udaipur, India); the average value of two measurements was used for further analyses. Body mass index (BMI, kg/m^2^) was calculated by dividing the weight (kg) by the square of the height (m^2^).

Self-reported smoking status—current, former, and never smoking—was obtained from the questionnaire, and the current status of non-smoking versus smoking was confirmed by urine cotinine levels (nmol/L) measured by immunoassay (Immulite 2000 Xpi; Siemens, NY, USA). One self-reported never smoker was re-assigned to current smoker based on a urine cotinine level of 16,909 nmol/L, higher than our cut-point for current smoking status of 283 nmol/L [32]. Smokers provided the duration (years) and amount (cigarette packs) of cigarette smoking. Pack-years were calculated by multiplying the number of smoked cigarette packs per day by the number of years smoked. Duration of smoking cessation (years) was reported by former smokers.

### Genomic DNA preparation and DNA methylation profiling

We used blood DNA samples from participants’ baseline visits for methylation profiling. The DNA quality was checked with a spectrophotometer (NanoDrop® ND-1000 UV-vis), and genomic DNA was diluted to 50 ng/μl using Quant-iT PicoGreen (Invitrogen, Carlsbad, CA, USA). Bisulfite-conversion using EZ DNA methylation kit (Zymo Research, Irvine, CA, USA) was carried out according to the manufacturer’s protocols.

The Infinium HumanMethylation450 BeadChip (Illumina, Inc., San Diego, CA, USA) was used for our genome-wide methylation profiling. The methylation value (*β*)—a ratio between methylated probe intensity and total probe intensity—is interpreted as the proportion of methylation and ranges between 0 (unmethylated) and 1 (methylated). The signal extraction and normalization using Beta MIxture Quantile dilation (BMIQ) [33] were conducted in ChAMP [34]. The ComBat [35] method was applied to adjust for batch effects. Cell-type composition was estimated by Houseman’s algorithm [36] in minfi [37]. Cytosine-phosphate-guanine (CpG) probe filtering criteria [38] were applied to eliminate sources of possible false positive results, excluding probes that had a detection *p* value above 0.01 in any sample; had a bead-count less than 3 in 5 % or more of samples; were non-CpG probes; or were non-specific probes [39]. To minimize the effects of extreme outliers at each probe on association results, methylation values outside three times the interquartile range (IQR) from the first and third quartiles were removed from the analyses. Of all beta values across all participants, 75,549 (0.19 %) were removed. Probes mapping to the X or Y chromosomes were removed [40]. Therefore, a total of 402,508 CpG probes were used in our EWAS.

### Statistical approach

We used methylation β values because they are more easily interpretable as methylation changes than *M* values [41]—the log2 ratio of methylated probe intensity and unmethylated probe intensity. To identify smoking-associated DMPs, we tested methylation levels (response) for association with smoking exposure status (predictor) using robust linear regression. We adjusted for COPD status because of the selection subjects and for age, sex, BMI, and estimated cell-type composition. Never smokers served as the reference group. The regression analysis and empirical Bayes approach was done using Linear Models for Microarray data (limma) [42]. For genome-wide significance, we set the threshold of false discovery rate (FDR) [43] adjusted *p* < 0.05. All results in this study are methylation differences in current smokers compared to never smokers unless otherwise noted.

In addition to association analyses at individual probes, we applied two different methods—DMRcate [44] and comb-p [45]—to detect regional methylation alterations. These methods can identify significant DMRs even when there is a lack of genome-wide significance at individual probe level. A DMR does not need to contain a DMP of genome-wide significance. DMRs were calculated based not on raw methylation data but the association results.

The DMR methods work in slightly different ways. DMRcate identifies DMRs using tunable kernel smoothing of association signals across the human genome. We used the “dmrcate” function in the DMRcate R package with an input file containing regression coefficients, standard deviations, and unadjusted *p* values for each probe from our EWAS of current smoking. In detail, DMRcate re-calculates *p* values at individual CpGs after modeling the Gaussian smoothing using Satterthwaite [46] method within a predefined bandwidth (the length of a distance), corrects *p* for multiple-testing, and combines information from nearby significant CpGs within the bandwidth. In contrast, comb-p identifies regional enrichments of low *p* values from unevenly spaced *p* values. It utilizes only unadjusted *p* values and chromosomal locations at each probe. It performs the Stouffer-Liptak-Kechris (slk) correction to adjust for adjacent *p* values after calculating auto-correlation, identifies regions of enrichment, generates Stouffer-Liptak region-corrected *p* values for each region, and performs Sidak [47] multiple-testing correction.

We defined significant DMRs (1) containing at least two probes, (2) combining information from probes residing within 1000 basepairs (bp), and (3) having multiple-testing corrected *p* < 0.01 (FDR for DMRcate and Sidak *p* for comb-p). These two values—the minimum number of CpGs in a region and the minimum length of a distance—were the defaults in DMRcate [48], so we used the same values for comb-p to compare results from two approaches. One DMR study using comb-p set the minimum number of probes to 2 and reported DMRs (Sidak *p* < 0.05) [49]. We used a more strict cutoff for multiple-testing correction (adjusted *p* < 0.01) for statistical significance because these methods have been updated and there is no consensus of the threshold. Relevant parameters for DMR calling can be found in Additional file 1: Table S2. We considered that the same region was identified as differentially methylated by the two methods if the start (bp) or end (bp) site was the same or a region identified by one of the two method resided inside a region identified by the other.

We evaluated whether the genome-wide significant (FDR < 0.05) differential methylation patterns seen in current smokers relative to never smokers were also seen in former compared to never smokers. Therefore, in the former smokers, we adjusted for 108 tests to determine look-up level replication (FDR < 0.05). In addition, we examined the dose-response relationships between methylation levels and quantitative indexes of smoking exposure: urine cotinine levels (nmol/L), pack-years in current smokers, and time since smoking cessation (years) in former smokers by using the Spearman correlation. For the dose-response analyses, we used nominal statistical significance (unadjusted *p* < 0.05) to report our findings.

We also examined the association with current smoking for the 192 CpGs reported more than once in the 16 published studies based on either Illumina Infinium HumanMethylation27 BeadChip or 450k array. Of these 192, 178 CpGs were checked for association after probe filtering in our data. The cutoff for statistical significance was set to FDR adjusted *p* < 0.05 after correcting for 178 tests.

All statistical analyses were performed in R (version 3.0.2) [50] except for comb-p [45]. The gene annotation for each probe was based on the manufacturer’s annotation file [51].

We used coMET [52] to visualize regional methylation patterns in the top four DMRs (adjusted *p* < 1.0E−10 at both analyses). In addition to gene names and regulatory elements of the region from ENSEMBLE, Digital DNaseI Hypersensitivity Clusters from ENCODE (DNase Cluster) and chromatin state segmentation by HMM from ENCODE/Broad (Broad ChromHMM) were added (Additional file 2: Figure S2).

### Enrichment and functional network analysis

We performed an enrichment analysis to examine whether the significant DMPs (FDR < 0.05) were over- or under-represented, compared to all probes from the 450k array, in several biological features from the Illumina annotation file. The hypergeometric test (two-sided doubling mid-p) was used for the evaluation of enrichments or depletions.

For biological insights into differential methylation changes in relation to current smoking, we implemented a functional network analysis. Genes annotated from selected DMPs (FDR < 0.10) were included in the analysis. We used a core analysis of Ingenuity Pathway Analysis (Ingenuity Systems, Inc., Redwood City, CA, USA).

### Transcriptome analysis: Asan Biobank

Transcriptome profiles from the lung tissues of 188 male smokers from the Asan Biobank were used in this analysis. Details of transcriptome profiles using RNA-seq (HiSeq 2000 system, Illumina, Inc., San Diego, CA, USA) have been published [30]. Data was available at NCBI Gene Expression Omnibus (GEO) (accession number of GSE57148). To exclude potential impact of extreme values, we filtered gene expression values outside of three times the IQR from the first and third quartiles of each gene transcript. Of all gene expression values across all participants, 35,607 (1.1 %) were removed. We calculated pack-years from duration (years) and amount (cigarette packs) of cigarette smoking.

To identify differentially expressed genes in relation to smoking intensity (pack-years), we applied a robust linear regression model and empirical Bayes approach by using limma [42]. For robust linear regression, gene expression levels were the response and pack-years the predictor. We presented nominally significant results to provide a clue to understand relationships between methylation in blood and gene expression in lung tissue.

## Results

The descriptive characteristics of the study populations are shown in Table 1. The study participants were aged 53 to 84 years. There were 39 never, 30 former, and 31 current smokers. Among the never smokers, 6 were male and 33 were female. The former smokers were all male. There was one female current smoker. Individuals diagnosed with COPD were represented in each smoking group as follows: 19 in never, 20 in former, and 21 in current smoking group. The average BMI was 23.2 kg/m^2^ for never smokers, 23.5 kg/m^2^ for former smokers, and 22 kg/m^2^ for current smokers. The duration of smoking cessation in former smokers ranged 7 to 40 years. There were no significant differences in age, BMI, and proportion of COPD cases across smoking groups in our EWAS data.

**Table 1:** Descriptive characteristics of the study population

Characteristics (mean ± standard deviation or *n* (%))	Genome-wide methylation analysis in blood DNA (the Korean COPD cohort)			Transcriptome analysis in lung tissue (Asan Biobank)
	Never smoker (*N* = 39)	Former smoker (*N* = 30)	Current smoker (*N* = 31)	
Male	6 (15.4)	30 (100)	30 (96.8)	188 (100)
Female	33 (84.6)	0 (0)	1 (3.2)	0 (0)
Age, years	72.9 ± 6.1	74.1 ± 7.4	71.5 ± 5.3	64.2 ± 8.7
Body mass index, kg/m^2^	23.2 ± 3.0	23.5 ± 2.7	22 ± 2.8	NA
Pack-year	NA^c^	28.9 ± 19.6	35.7 ± 19.1	42.0 ± 20.6
Duration of smoking cessation, years	NA	17.6 ± 7.5	NA	NA
Urine cotinine, nmol/L	88.4 ± 3.2^d^	167.6^e^	29421 ± 21947	NA
Undetectable^a^	36 (92.3)	29 (96.7)	0 (0)	NA
COPD^b^	19 (48.7)	20 (66.7)	21 (67.7)	98 (51.9)

^a^Urine cotinine levels ≤56.8 nmol/L are marked as “undetectable” from the measurement using IMMULITE 2000 Immunoassay System (Siemens Healthcare Diagnostics Inc., Tarrytown, NY, USA)

^b^Chronic obstructive pulmonary disease

^c^Not available

^d^Urine cotinine levels in three never smokers were detectable

^e^Urine cotinine level in only one former smoker was detectable and the level was 167.6 nmol/L

We identified 108 significant DMPs in current smokers compared to never smokers (FDR < 0.05) (Table 2, Additional file 3: Table S1, and Additional file 4: Table S3). Of these, nine were significant after Bonferroni correction (unadjusted *p* < 1.2E−07 correcting for 402,508 tests). Of the FDR-significant DMPs, 93 of these were novel and 15 were previously reported in EWASs of smoking. Decreased methylation in current smokers was observed at 85 % of the significant DMPs. The methylation differences between current and never smokers at significant CpGs ranged from −20.3 to 15.6 %. Among the top five probes, the most highly statistically significant was a CpG well-known for its association with smoking: cg05575921 (FDR = 2.6E−07) in aryl-hydrocarbon receptor repressor (*AHRR*). Among the remaining four probes in the top five, three were novel—cg10664184 (FDR = 1.80E−05) in *DDA1*; cg20723792 (FDR = 6.40E−05) in *FAM53B*; and cg24780263 (FDR = 0.001) in *ALDOA*—except for cg05951221 (FDR = 8.50E−04) located 12,850 base pair (bp) apart from *ALPPL2*. At five loci, more than one DMP at genome-wide significance was identified: *AHRR* (3 probes), 2q37.1 near *ALPPL2* (2 probes), *MYO1G* (2 probes), *NKX2-3* (2 probes), and *FAM82A2* (2 probes). The genomic inflation factor (lambda) was 1.25. Manhattan plot and QQ plot are provided (Additional file 5: Figure S1).

**Table 2:** Top 30 CpGs differentially methylated in blood DNA in relation to current smoking compared to never smoking (FDR < 0.05, ordered by chromosomal location)

Chr^a^	Gene	Distance to gene^b^	Probe	Position^c^	Coef^d^	SE^e^	*P* ^f^
2	*CCDC104*		cg21597209	55746709	−0.009	0.002	6.2E−07
	*DGUOK*		cg19394739	74154363	−0.012	0.002	3.5E−07
	*CLASP1*		cg22346073	122402890	−0.056	0.010	5.1E−08
	*SATB2*		cg21136715	200322252	−0.035	0.006	2.1E−07
	*ALPPL2*	12,850	cg05951221^g^	233284402	−0.088	0.014	8.4E−09
3	*GPR15*		cg19859270^g^	98251294	−0.027	0.005	1.0E−07
5	*AHRR*		cg05575921^g^	373378	−0.203	0.025	6.5E−13
			cg25648203^g^	395444	−0.079	0.015	6.2E−07
	*LINC01019*	−239,389	cg11405538	3177877	0.124	0.022	1.3E−07
	*SOX30*		cg06995810	157079468	0.048	0.009	1.0E−06
7	*TSPAN13*		cg05848863	16794078	−0.024	0.004	3.6E−07
	*PLEKHA8*		cg09762120	30108301	0.040	0.007	2.8E−08
	*ADCYAP1R1*		cg20165074	31091813	−0.008	0.002	6.7E−07
10	*FAM53B*		cg20723792	126360669	−0.097	0.014	4.8E−10
11	*IRF7*		cg27271532	612762	−0.035	0.006	3.8E−07
	*E2F8*		cg15604507	19263433	−0.021	0.004	5.7E−07
	*CCND1*		cg09520904	69462943	−0.036	0.007	7.5E−07
	*DIXDC1*		cg11471799	111807548	−0.023	0.004	6.2E−07
12	*CDK2AP1*		cg13421247	123756945	−0.058	0.011	9.8E−07
14	*CFL2*	−44,147	cg23429457	35135441	−0.040	0.007	2.0E−07
	*EXOC3L4*	−20,369	cg04884342	103546112	0.020	0.004	5.6E−07
15	*CALML4*		cg00388154	68498857	−0.058	0.011	2.9E−07
	*CORO2B*		cg18765659	69018349	−0.053	0.010	7.4E−07
	*TLE3*		cg06730438^h^	70355664	−0.016	0.003	4.9E−07
16	*ALDOA*		cg24780263	30064201	−0.011	0.002	1.8E−08
	*KIAA0182*		cg26723054	85650522	−0.038	0.007	7.2E−07
19	*F2RL3*		cg03636183^g^	17000585	−0.128	0.021	2.0E−08
	*DDA1*		cg10664184	17420304	−0.028	0.004	9.2E−11
	*CD33*		cg06861672	51727798	−0.036	0.007	3.3E−07
21	*MIR155HG*		cg03872783	26934885	−0.008	0.001	9.7E−07

^a^Chromosome

^b^Distance to transcription start site of the mapped gene (basepair)

^c^Physical position (basepair, National Center for Biotechnology Information human reference genome assembly Build 37.3)

^d^Regression coefficient from statistical model

^e^Standard error of regression coefficient

^f^Statistical significance from statistical model

^g^Probe identified in previous epigenome-wide association studies (EWASs) of smoking

^h^Probe mapped to genes identified in previous EWASs of smoking

DMPs ordered by p values can be found in Additional file 10

For our 108 significant DMPs, we found enrichment of probes mapping to CpG island shores (35 versus 23 % overall from the array, *p* = 0.002) and enhancer (29 versus 21 % overall from the array, *p* = 0.04). No significant over- or under-representation of probes in promoter-associated regions (19 versus 19 % overall, *p* > 0.05) or DNase hypersensitivity sites (18 versus 12 % overall, *p* > 0.05) were detected.

From the two different DMR analyses, we discovered 249 significant (FDR < 0.01) DMRs from DMRcate, 102 significant (Sidak *p* < 0.01) DMRs from comb-p, and 87 significant based on both approaches (Table 3). Of these 87 significant using both methods, 66 regions were novel, meaning never reported in previous EWASs of smoking in adults, including 7 that contained one of our genome-wide significant individual DMPs. Among those 87 DMRs, the most significant one (chromosome:start position-end position) from DMRcate was chr5:373378–374425 (FDR = 4.6E−17) in *AHRR* and this region contains five probes—cg05575921, cg22103736, cg08714121, cg04141806, and cg22356527—including our top-ranked DMP. *AHRR* differential methylation was also observed from comb-p with two probes—cg05575921 and cg22103736—in slightly shorter length (chr5:373378–373887; Sidak *p* = 4.8E−05) than that from DMRcate. The most significant DMR overall from comb-p was chr6:149805995–149806732 (Sidak *p* = 1.9E−14) in *ZC3H12D* and the exact same region, meaning the same start, end, and number of probes, was also observed from DMRcate (FDR = 2.3E−15) (Table 3). This region did not contain a genome-wide significant DMP. Among novel DMRs, the top two regions from both analyses were chr4:81117647–81119473 (FDR = 6.7E−13 from DMRcate; Sidak *p* = 2.9E−13 from comb-p) at *PRDM8* including 11 probes and chr4:103940711–103941300 (FDR = 6.8E−14 from DMRcate; Sidak *p* = 2.7E−10 from comb-p) at *SLC9B1* including 11 probes. Details of the top five DMRs from each software are in Additional file 6: Table S4. Those regions contain either one or two highly significant CpGs or tightly spaced CpGs of nominal statistical significance. The average (standard deviation, SD) of distances of nearby CpGs in those regions was 147 (153) bp for DMRcate and 158 (169) bp for comb-p.

**Table 3:** Differentially methylated regions in blood DNA in relation to current smoking compared to never smoking (multiple-testing corrected *p* < 0.01 at DMRcate and comb-p, ordered by chromosomal location)

Chr^a^	Gene	Distance to gene^b^	DMRcate				Comb-p				Minimum *P* ^i^
			Start (bp^c^)	End (bp)	FDR^d^	#CpGs^e^	Start (bp)	End (bp)	Sidak P^f^	#CpGs	
1	*MXRA8*	−812	1286917	1287259	0.002	2(2)			0.002		2.2E−04
	*CASZ1*	−600	10695686	10696066	8.7E−04	2(2)			0.009		1.5E−05
	*AHDC1*		27929092	27929260	2.2E−04	2(2)			0.006		1.5E−04
	*NT5C1A*		40137636^g^	40138402	3.2E−06	6(3)			0.001		5.5E−06
	*ACOT11* ^h^	−58,441	54954187	54955366	0.002	7(4)	54953632		0.009	8(4)	6.1E−04
	*GNG12* ^h^		68298816^g^	68299511	7.0E−07	7(5)	68299057		0.001	6(5)	1.4E−06
	*GFI1* ^h^		92946700	92947961	1.1E−04	6(4)			1.2E−04		8.1E−05
	*SPAG17*		118727658^g^	118728226	1.3E−04	10(2)			0.005		7.1E−06
	*ZNF697*		120173989	120174570	0.006	4(4)		120174873	0.006	6(4)	0.002
	*GALNT2*		230415343	230416101	0.002	6(3)	230414987	230417096	1.2E−04	12(4)	0.005
	*SCCPDH*	−26,962	246859889	246860416	7.0E−04	5(4)			2.6E−04		0.002
2	*PAX8*		113992762	113993313	0.005	8(6)			0.004		0.011
	*ALPPL2* ^h^	11,458	233283010^g^	233285607	8.0E−15	8(5)			1.5E−13		8.4E−09
	*SNED1* ^h^		241975756	241976244	1.9E−06	4(4)			3.8E−06		1.4E−04
3	*KRBOX1*	11	42977777	42978180	9.7E−04	7(5)			0.003		4.1E−04
	*GPR15* ^h^		98250723^g^	98251294	6.2E−07	2(1)	98249859		6.2E−04	4(2)	1.0E−07
	*ZBTB38*		141086820	141087363	0.006	6(4)			0.005		0.005
	*LPP* ^h^		187870621	187871538	1.5E−05	11(5)			0.001		1.1E−04
	*C3orf43*	21,882	196255632	196256223	9.7E−04	5(3)			0.004		1.8E−04
4	*PCGF3*		737005	738199	0.002	8(2)	736328		0.001	12(4)	2.5E−05
	*FGFRL1*	−1776	1003208	1003834	1.5E−04	3(2)			0.002		2.0E−04
	*PRDM8*		81117647^g^	81119473	6.7E−13	11(10)			2.9E−13		6.7E−06
	*NHEDC1*		103940711	103941300	6.8E−14	11(10)			2.7E−10		6.2E−05
	*CFI*		110724358	110724834	0.006	2(2)			0.009		4.4E−04
5	*AHRR* ^h^		373378^g^	374425	4.6E−17	5(2)		373887	4.8E−05	2(1)	6.5E−13
			392920^g^	393366	5.8E−08	3(3)			3.9E−08		4.7E−06
	*LPCAT1*		1494980	1495356	0.001	5(4)			0.003		0.001
	*LINC01019*	−236,319	3180918	3180947	0.006	2(2)		3182108	6.0E−04	5(4)	5.7E−04
	*FLJ44606*		126408756	126409553	7.0E−07	13(11)			1.9E−06		0.001
	*ADAMTS2*		178548229	178548700	0.002	3(3)			0.003		8.5E−04
6	*IER3* ^h^	9104	30720080	30720491	1.2E−06	8(4)			0.002		1.7E−05
	*LY6G6E*		31683051	31683352	5.4E−05	6(5)			1.2E−04		0.002
	*HLA-DPB1*		33047944	33049505	2.5E−09	20(15)			4.8E−08		0.002
	*SYNGAP1* ^h^		33400477	33401542	6.9E−06	9(7)	33400021		2.2E−05	10(7)	2.7E−04
	*CRISP2*		49681178	49681774	5.5E−06	9(8)			5.5E−06		1.8E−04
	*UTRN*	−4373	144607399	144608500	0.004	7(4)	144607074		0.010	8(4)	2.6E−04
	*ZC3H12D* ^h^		149805995	149806732	2.3E−15	10(10)			1.9E−14		8.7E−05
	*TIAM2* ^h^		155537595	155538155	1.6E−05	8(5)			3.7E−05		7.6E−04
	*THBS2*		169653612	169654719	9.5E−04	11(4)		169654842	7.0E−04	12(4)	5.3E−04
7	*GNA12* ^h^		2768988	2770410	4.7E−06	5(5)	2769253		7.4E−05	4(4)	3.0E−05
	*TRG-AS1* ^h^	−29,710	38350464	38351468	2.0E−06	7(6)			1.1E−05		1.7E−04
	*MYO1G* ^h^		45001765^g^	45002919	5.5E−14	6(5)			5.7E−09		2.7E−06
	*INSIG1*	61,195	155150681	155151427	0.007	4(3)			0.002		0.003
8	*DEFA4*		6795162	6796618	2.0E−04	4(4)	6794872		1.7E−05	5(4)	4.0E−05
	*EPB49* ^h^		21915184	21915510	0.004	2(2)	21914287	21916853	5.3E−05	11(6)	0.002
	*TRAPPC9*		141057285	141057827	3.7E−06	5(5)			2.1E−06		2.0E−04
	*GLI4*		144358043	144359316	0.001	5(5)			1.5E−05		0.002
9	*CD72*		35609853	35610380	0.002	2(2)			0.007		1.1E−04
	*CIZ1*		130955135	130956057	0.001	4(3)		130955436	0.004	3(3)	0.001
10	*SNCG*		88717926	88718393	5.5E−04	5(5)			3.8E−04		0.003
	*SLC16A12* ^h^		91296252	91296457	1.6E−04	3(3)			0.004		4.4E−04
	*LGI1*		95517382	95517895	6.3E−04	7(4)			0.002		5.6E−04
	*NKX2-3*	−4844	101287381^g^	101287846	8.2E−06	5(3)			1.3E−04		7.4E−06
	*GRK5*		121171859	121172898	6.4E−04	5(4)			2.4E−04		4.1E−04
11	*C11orf21* ^h^		2321770	2322674	1.2E−05	18(7)		2323938	1.3E−04	33(8)	5.9E−04
	*C11orf41*		33562503	33563377	7.0E−04	4(4)		33563946	2.3E−04	5(4)	5.4E−04
	*NEAT1* ^h^	4664	65194933	65196227	2.2E−05	7(7)		65196696	3.0E−05	10(7)	4.9E−04
	*ACY3*		67418045	67418405	1.1E−09	12(11)			8.7E−08		1.3E−04
	*CCND1*		69462660^g^	69463323	2.4E−06	6(3)			1.7E−04		7.5E−07
	*AMICA1* ^h^		118084920	118085736	0.005	4(4)			0.002		0.003
12	*IFFO1*		6657744	6658945	2.7E−04	10(5)		6659524	2.2E−04	12(5)	8.1E−05
	*MGP*		15038440	15039432	9.5E−04	4(3)			3.5E−05		9.3E−05
	*KRT7*		52638005	52638592	0.002	3(2)			0.005		1.5E−04
	*ZNF385A*		54778312	54779175	0.002	4(3)			0.008		0.001
	*RP11-474D1.3*	36,620	130554977	130555091	1.8E−04	3(3)			9.4E−04		1.7E−04
	*STX2*	−73,033	131199848	131201112	7.2E−04	10(4)	131198873	131201268	0.008	12(5)	6.5E−05
14	*LGMN*		93170710	93170970	0.002	3(3)			0.008		6.6E−05
	*EVL*		100610071	100610667	9.8E−05	6(4)			1.9E−04		4.0E−04
	*RIN3*		92981121	92981666	1.6E−05	3(3)			2.1E−05		1.8E−04
15	*CALML4*		68498251^g^	68499367	2.6E−06	5(2)	68497992		0.002	6(2)	2.9E−07
16	*PRR25*		854168	854640	0.002	4(3)		855449	0.002	6(4)	7.7E−04
	*BCL7C*		30906810	30907246	0.001	2(2)		30907560	8.0E−04	3(3)	9.0E−04
17	*ALOX15B*		7942137	7942743	1.1E−04	6(5)			2.4E−04		3.9E−04
	*NTN1*		9018806	9019336	2.0E−05	5(4)			5.5E−04		5.3E−04
	*SLFN12L*	−13,916	33787402	33788026	0.003	4(4)			0.001		8.8E−04
	*CYB561*		61511069	61511829	4.9E−04	4(4)			9.3E−05		5.2E−04
	*CCDC57*		80076338	80076378	1.1E−04	2(2)			0.002		2.2E−05
	*FOXK2*		80545020^g^	80545869	8.1E−08	11(6)			2.6E−04		5.5E−06
	*TBCD*		80870107	80870923	0.001	5(3)		80871405	0.002	7(4)	1.8E−04
18	*C18orf1*		13611370	13611824	0.007	6(4)			0.009		0.003
19	*GNG7*		2543602	2544100	0.008	5(2)	2542837		0.002	6(3)	6.4E−04
	*MAN2B1*		12758416	12759546	0.004	7(4)			0.001		0.002
	*LAIR1*		54876446	54876795	1.8E−04	5(4)			8.1E−04		2.3E−04
20	*C20orf27*		3745817	3746315	0.002	2(2)			0.004		8.8E−05
22	*SYNGR1*		39759864^g^	39760267	1.2E−07	5(5)			1.2E−06		2.5E−06
	*SHISA8*	−978	42304331	42304580	1.4E−04	2(2)			6.9E−04		2.3E−05
	*ODF3B*		50970943	50971140	4.2E−04	3(3)			0.002		1.6E−04

Empty cells in “Start,” “End,” and “#CpGs” for comb-p represent the same regional information compare to results in DMRcate. D﻿MRs ordered by p values can be found in Additional file 11

^a^Chromosome

^b^Minimum distance to transcription start site of the mapped gene (basepair)

^c^Physical position (basepair, National Center for Biotechnology Information human reference genome assembly Build 37.3)

^d^False discovery rate

^e^Number of probes in the region (number of CpGs of nominal statistical significance)

^f^ *P* of Sidak multiple-testing correction

^g^Region including significant (FDR <0.05) differentially methylated probes from our epigenome-wide association study (EWAS)

^h^Gene identified in previous EWASs of smoking

^i^Minimum *p* values among unadjusted *p* values of CpGs in each region

Among the 108 significant DMPs from the comparison of current to never smokers, 104 were also significant in the former to never smoker comparison (FDR <0.05, look-up level replication) and had effects in the same direction (Additional file 7: Table S5). The attenuation in effect size in former compared with current smokers ranged from −12.3 to 4.3 %. The top-ranked DMP in former smokers compared to never smokers was cg20723792 (FDR = 1.3E−2) in *FAM53B* at which no relationship with smoking exposures in terms of DNA methylation has been previously reported.

We examined dose-response relationships between methylation levels and quantitative measures of smoking exposure (urine cotinine levels and pack-years in current smokers and duration of smoking cessation in former smokers) for the 108 significant DMPs identified in our EWAS of current smoking (Table 4). There was no significant finding after FDR multiple-testing correction. Urine cotinine levels were positively correlated at nominal levels of significance (uncorrected *p* < 0.05) with methylation levels at a probe in *MTNR1A* and negatively correlated with methylation levels at five probes from five different loci: *GNG12*; *GPR15*; *AHRR*; *FAM82A2*; and *F2RL3*. Pack-years in current smokers showed positive correlation at five loci and negative correlation with methylation levels at one locus. Duration of smoking cessation in former smokers was positively correlated at nominal significance (*p* < 0.05) with methylation levels at seven loci and negatively correlated with methylation at one locus.

**Table 4:** CpGs differentially methylated in relation to smoking status also related to quantitative measures of smoking (*p* correlation <0.05, ordered by chromosomal location)

Chr^a^	Gene	Distance to gene^b^	Probe	Epigenome-wide association study		ρ^d^	P_ρ_
				Coef^c^	*p*		
Urine cotinine in current smokers (*N* = 31)							
1	*GNG12*		cg25189904^e,f^	−0.134	1.4E−06	−0.40	0.027
3	*GPR15*		cg19859270^e,f^	−0.027	1.0E−07	−0.56	0.001
4	*MTNR1A*		cg22261866	−0.063	1.6E−06	0.37	0.041
5	*AHRR*		cg05575921^e,f^	−0.203	6.5E−13	−0.43	0.016
15	*FAM82A2*		cg19440278	0.007	7.0E−06	−0.43	0.016
19	*F2RL3*		cg03636183^e,f^	−0.128	2.0E−08	−0.56	0.001
Pack-year in current smokers (*N* = 31)							
1	*NT5C1A*		cg00990022	−0.04	5.5E−06	0.39	0.036
6	*ZBTB9*		cg03945003	−0.023	3.9E−06	0.40	0.031
10	*JAKMIP3*		cg19134728^e^	−0.023	1.2E−05	0.37	0.045
11	*HPX*		cg25426350	−0.03	2.5E−06	0.44	0.016
11	*CCND1*		cg09520904	−0.036	7.5E−07	−0.44	0.015
21	*RNF160*		cg13662262	−0.01	9.2E−06	0.44	0.015
Time since quit smoking in former smokers (*N* = 30)							
1	*IFI16*	−9970	cg19707735	−0.035	1.0E−04	0.47	0.009
2	*CLASP1*		cg22346073	−0.052	8.0E−07	0.43	0.017
3	*ARHGEF3*		cg25799109^e^	−0.076	4.4E−05	−0.44	0.016
3	*KTELC1*		cg16958524	−0.029	6.9E−06	0.39	0.033
5	*SPEF2*		cg08534016	−0.050	0.001	0.42	0.021
6	*ACOT13*	16438	cg09447457	−0.010	1.2E−05	0.39	0.034
9	*BSPRY*		cg02003202	−0.049	9.5E−06	0.44	0.015
15	*FAM82A2*		cg21580007	−0.049	9.0E−04	0.47	0.009

Results for current and former smokers showed regression coefficients and *p* values from EWAS for current and former smokers, respectively

^a^Chromosome

^b^Distance to transcription start site of the mapped gene (basepair, based on National Center for Biotechnology Information human reference genome assembly Build 37.3)

^c^Regression coefficient from statistical model

^d^Spearman correlation (rho) was used for urine cotinine and pack-years in current smokers and time since quit smoking in former smokers. The methylation values were adjusted for age, sex, body mass index, chronic obstructive pulmonary disease status, and estimated cell composition

^e^Probe identified in previous epigenome-wide association studies (EWASs) of smoking

^f^Probe identified in one previous EWAS of serum cotinine

Our analysis of differential gene expression in lung tissue was conducted in 188 male smokers from a separate study, the Asan Biobank. The average age was 64.2 (SD = 8.7) years and average pack-years was 42.0 (SD = 20.6) (Table 1). Of the 174 genes to which the 108 DMPs or 87 DMRs that were significantly differentially methylated were annotated, we had gene transcript profiles for 143. Of these, 20 genes, annotated from 17 DMPs or eight DMRs, showed nominally significant differential gene expression profiles (*p* < 0.05) in relation to pack-years (Table 5). Fourteen of the 20 genes were novel loci for effects of smoking on methylation and six—*GPR15*, *AHRR*, *ELMO1*, *SNED1*, *LPP*, and *GNA12*—were previously reported in EWASs of smoking. No significant results were observed after FDR multiple-testing correction.

**Table 5:** Differential methylation in relation to current smoking for genes with transcripts differently expressed (*p* < 0.05) in relation to smoking pack-years (ordered by chromosomal location)

Differentially methylated probes in relation to current smoking compared to never smoking (the Korean COPD cohort)						Gene (distance to gene^c^)	Differentially expressed genes in relation to pack-years in lung tissue (Asan Biobank)		
Differentially methylated probe									
Chr^a^	Probe	Coef ^b^	*P*	Genomic features	CpG island		Transcript	Coef	*P*
1	cg20388635	−0.013	1.3E−05	TSS200, promoter	Island	*YTHDF2*	NM_001173128	−0.019	0.047
2	cg22346073	−0.056	5.1E−08	5′UTR	Shelf	*CLASP1*	NM_015282	0.019	7.2E−04
	cg19394739	−0.012	3.5E−07	Body, promoter	Shore	*DGUOK*	NM_080916	−0.079	0.003
	cg09059267	−0.099	4.2E−06		Island	*DNPEP* (−15098)	NM_012100	−0.021	0.037
3	cg01870865	−0.045	1.0E−05	TSS200, promoter		*TREX1*	NM_033629	−0.018	0.023
	cg19859270^d^	−0.027	1.0E−07	1st exon		*GPR15* ^e^	NM_005290	0.013	3.0E−04
5	cg05575921^d^	−0.203	6.5E−13	Body, enhancer	Shore	*AHRR* ^e^	NM_001242412	0.004	0.047
	cg14817490^d^	−0.078	4.7E−06	Body, promoter, DHS					
	cg25648203^d^	−0.079	6.2E−07	Body, enhancer, DHS					
6	cg23164938	−0.016	9.5E−06	TSS1500	Shore	*ESR1*	NM_000125	0.005	0.012
7	cg05383910	−0.042	2.1E−06	5′UTR, enhancer		*ELMO1* ^e^	NR_038121	0.017	0.031
	cg20663219	−0.054	9.4E−06	Body, DHS	Shelf	*STX1A*	NM_001165903	−0.003	0.045
10	cg20723792	−0.097	4.8E−10	Body, enhancer, DHS		*FAM53B*	NM_014661	0.009	0.042
11	cg25426350	−0.030	2.5E−06	TSS200		*HPX*	NM_000613	−0.003	0.024
13	cg17058676	−0.028	2.5E−06	Body	Shore	*CENPJ*	NM_018451	0.003	0.035
14	cg16579351	−0.017	1.2E−05	Body		*BRF1*	NM_001242788	−0.012	0.041
17	cg13521620	−0.052	1.2E−05	5′UTR	Shore	*YPEL2*	NM_001005404	0.019	0.023
Differentially methylated region									
Chr	Region	#CpGs	FDR	Genomic features	CpG island				
1	230415343–230416101	6	0.002	3′UTR	Island, shore	*GALNT2*	NM_004481	0.033	0.018
2	241975756–241976244	4	1.9E−06	Body, promoter, DHS	island	*SNED1* ^e^	NM_001080437	0.017	0.014
3	98250723–98251294	2	6.2E−07	TSS200, 1st exon		*GPR15* ^e^	NM_005290	0.013	3.0E−04
3	187870621–187871538	11	1.5E−05	TSS1500, TSS200	Shore, island	*LPP* ^e^	NM_005578	0.019	0.018
5	373378–374425	5	4.6E−17	Body, enhancer	Shore, island	*AHRR* ^e^	NM_001242412	0.004	0.047
5	392920–393366	3	5.8E−08	Body, promoter, DHS					
7	2768988–2770410	5	4.7E−06	3′UTR, enhancer		*GNA12* ^e^	NM_007353	0.022	0.019
17	61511069–61511829	4	4.9E−04	3′UTR, body, enhancer	Shore, island	*CYB561*	NM_001017916	−0.056	0.005

Genomic features were based on Illumina’s Annotation file and those for DMRs were based on CpGs at start and end position of each region. Categories for the features includes (1) Body, gene body; (2) 5′UTR, 5 prime untranslated region; (3) 3′UTR, 3 prime untranslated region; (4) TSS200, 200 basepair within transcription start site; (5) TSS1500, 1500 basepair within transcription start site; and (6) DHS, DNase I hypersensitivity site

^a^Chromosome

^b^Regression coefficient from statistical model

^c^Distance to transcription start site of the mapped gene (basepair, National Center for Biotechnology Information human reference genome assembly Build 37.3)

^d^Probe identified in previous epigenome-wide association studies (EWASs) of smoking

^e^Gene identified in previous EWASs of smoking

In current smokers compared to never smokers, there were lower methylation levels at 17 DMPs (Table 5). Of those, four CpGs were located in enhancer regions and their corresponding lung tissue gene expression values were positively associated with pack-years in smokers, regardless of whether or not they were located in a CpG island. Four of the 17 were at DNase I hypersensitivity sites (DHS). Three of these were outside of CpG islands and showed a positive association with pack-years in smokers. The remaining site, located on a shelf region of a CpG island, was negatively associated. At four promoter-associated CpGs, we did not find any relationships between methylation levels and gene expression values.

Our functional network mapping involving 221 genes annotated from probes in our EWAS (FDR < 0.10) identified four overrepresented pathways (Additional file 8: Table S6). Top three networks were “gene expression, cellular movement, and embryonic movement,” “cancer, cellular development, organismal injury, and abnormalities,” and “hematological, metabolic, and cardiovascular disease.”

From a replication look-up of 178 CpGs, selected based on significant findings in at least two published EWASs of smoking, we confirmed differential methylation at 70 CpGs (Table 6). Of these, all CpGs showed same direction of association compared to that in previous reports. Among these 178 probes from previous EWASs, 83 (47 %) showed nominal (*p* < 0.05) association in our analysis of current smokers which is much higher expected by chance (Kolmogorov *p* < 2.2E−16). There were also significant differential methylation changes in former smokers at 24 CpGs in 17 loci (Table 6).

**Table 6:** Look-up in the Korean COPD cohort of CpGs reported at least two epigenome-wide association studies (70 CpGs at FDR^g^ < 0.05, ordered by chromosomal location)

Chr^a^	Gene	Distance to gene^b^	Probe	Coef^c^	*P* ^d^	References^e^
1	*GNG12*		cg25189904^f^	−0.134	1.4E−06	Guida et al. 2015 [12]; Besingi and Johansson 2014 [14]; Elliott et al. 2014 [16]; Zeilinger et al. 2013 [22]; Tsaprouni et al. 2014[18]; Zhu et al. 2016 [26].
			cg26764244	−0.055	0.010	Guida et al. 2015 [12]; Harlid et al. 2014[17].
	*GFI1*		cg12876356^f^	−0.049	8.9E−04	Guida et al. 2015 [12]; Besingi and Johansson 2014 [14]; Dogan et al. 2014[15]; Zeilinger et al. 2013 [22]; Zhu et al. 2016 [26].
			cg18316974	−0.014	0.006	Guida et al. 2015 [12]; Besingi and Johansson 2014 [14]; Zeilinger et al. 2013 [22].
			cg09935388	−0.106	8.1E−05	Guida et al. 2015 [12]; Besingi and Johansson 2014 [14]; Dogan et al. 2014[15]; Elliott et al. 2014 [16]; Zeilinger et al. 2013 [22]; Zhu et al. 2016 [26].
	*AVPR1B*		cg08709672^f^	−0.058	1.1E−06	Guida et al. 2015 [12]; Besingi and Johansson 2014 [14]; Elliott et al. 2014 [16]; Zeilinger et al. 2013 [22]; Zhu et al. 2016 [26].
			cg20295214	−0.068	3.5E−04	Guida et al. 2015 [12]; Besingi and Johansson 2014 [14]; Tsaprouni et al. 2014[18]; Zeilinger et al. 2013 [22].
	*PSEN2*	−55213	cg03547355	−0.034	0.016	Guida et al. 2015 [12]; Tsaprouni et al. 2014[18]; Zeilinger et al. 2013 [22].
2	*LINC00299*	195809	cg23079012^f^	−0.023	3.8E−04	Besingi and Johansson 2014 [14]; Tsaprouni et al. 2014[18]; Zeilinger et al. 2013 [22]; Zhu et al. 2016 [26].
	*NFE2L2*		cg26271591^f^	−0.061	8.3E−04	Guida et al. 2015 [12]; Besingi and Johansson 2014 [14]; Zeilinger et al. 2013 [22].
	*GPR55*		cg19827923	−0.022	0.012	Guida et al. 2015 [12]; Zhu et al. 2016 [26].
	*ALPP*		cg23667432	−0.027	0.013	Guida et al. 2015 [12]; Zeilinger et al. 2013 [22].
	*ECEL1P2*	−90	cg27241845	−0.081	4.1E−04	Guida et al. 2015 [12]; Besingi and Johansson 2014 [14]; Zeilinger et al. 2013 [22]; Tsaprouni et al. 2014[18].
	*ALPPL2*	11777	cg03329539^f^	−0.064	3.9E−05	Guida et al. 2015 [12]; Besingi and Johansson 2014 [14]; Dogan et al. 2014[15]; Elliott et al. 2014 [16]; Tsaprouni et al. 2014[18]; Zeilinger et al. 2013 [22]; Zhu et al. 2016 [26].
		12850	cg05951221^f^	−0.088	8.4E−09	Allione et al. 2015 [11]; Guida et al. 2015 [12]; Besingi and Johansson 2014 [14]; Dogan et al. 2014[15]; Harlid et al. 2014[17]; Elliott et al. 2014 [16]; Tsaprouni et al. 2014[18]; Shenker et al. 2013[19]; Zeilinger et al. 2013 [22]; Zhu et al. 2016 [26].
		13382	cg01940273	−0.090	1.4E−06	Allione et al. 2015 [11]; Guida et al. 2015 [12]; Besingi and Johansson 2014 [14]; Dogan et al. 2014[15]; Elliott et al. 2014 [16]; Tsaprouni et al. 2014[18]; Shenker et al. 2013[19]; Zeilinger et al. 2013 [22]; Zhu et al. 2016 [26].
		13737	cg13193840^f^	−0.027	1.1E−04	Guida et al. 2015 [12]; Tsaprouni et al. 2014[18]; Zeilinger et al. 2013 [22]; Zhu et al. 2016 [26].
	*SNED1*		cg26718213	0.091	0.005	Guida et al. 2015 [12]; Besingi and Johansson 2014 [14].
3	*GPX1*		cg18642234	−0.042	0.005	Guida et al. 2015 [12]; Zeilinger et al. 2013 [22]; Zhu et al. 2016 [26].
	*GPR15*		cg19859270	−0.027	1.0E−07	Guida et al. 2015 [12]; Besingi and Johansson 2014 [14]; Dogan et al. 2014[15]; Harlid et al. 2014[17]; Elliott et al. 2014 [16]; Tsaprouni et al. 2014[18]; Sun et al. 2013[20]; Zeilinger et al. 2013 [22]; Wan et al. 2012[24]; Breitling et al. 2011[25]; Zaghlool et al. 2015[13]; Zhu et al. 2016 [26].
	*CPOX*		cg02657160	−0.030	1.8E−04	Guida et al. 2015 [12]; Besingi and Johansson 2014 [14]; Dogan et al. 2014[15]; Harlid et al. 2014[17]; Tsaprouni et al. 2014[18]; Zeilinger et al. 2013 [22].
5	*AHRR*		cg11554391	−0.043	2.5E−04	Guida et al. 2015 [12]; Zeilinger et al. 2013 [22]; Zhu et al. 2016 [26].
			cg12806681^f^	−0.015	0.009	Guida et al. 2015 [12]; Besingi and Johansson 2014 [14]; Dogan et al. 2014[15]; Zeilinger et al. 2013 [22]; Zhu et al. 2016 [26].
			cg23916896^f^	−0.063	0.006	Guida et al. 2015 [12]; Dogan et al. 2014[15]; Zeilinger et al. 2013 [22]; Zhu et al. 2016 [26].
			cg01899089^f^	−0.054	0.003	Guida et al. 2015 [12]; Besingi and Johansson 2014 [14]; Dogan et al. 2014[15]; Zeilinger et al. 2013[22].
			cg05575921^f^	−0.203	6.5E−13	Allione et al. 2015 [11]; Guida et al. 2015 [12]; Besingi and Johansson 2014 [14]; Dogan et al. 2014[15]; Harlid et al. 2014[17]; Elliott et al. 2014 [16]; Tsaprouni et al. 2014[18]; Shenker et al. 2013[19]; Zeilinger et al. 2013 [22]; Zaghlool et al. 2015[13]; Philibert et al. 2012[23]; Philibert et al. 2013[21]; Zhu et al. 2016 [26].
			cg14817490	−0.078	4.7E−06	Guida et al. 2015 [12]; Besingi and Johansson 2014 [14]; Elliott et al. 2014 [16]; Tsaprouni et al. 2014[18]; Zeilinger et al. 2013 [22]; Zaghlool et al. 2015[13]; Zhu et al. 2016 [26].
			cg17287155	−0.023	1.8E−04	Guida et al. 2015 [12]; Dogan et al. 2014[15]; Zhu et al. 2016 [26].
			cg04551776	−0.038	1.3E−04	Guida et al. 2015 [12]; Elliott et al. 2014 [16]; Zhu et al. 2016 [26].
			cg25648203	−0.079	6.2E−07	Allione et al. 2015 [11]; Guida et al. 2015 [12]; Besingi and Johansson 2014 [14]; Dogan et al. 2014[15]; Elliott et al. 2014 [16]; Zeilinger et al. 2013 [22]; Tsaprouni et al. 2014[18]; Zhu et al. 2016 [26].
			cg24090911	−0.039	0.009	Guida et al. 2015 [12]; Tsaprouni et al. 2014[18]; Zeilinger et al. 2013 [22]; Zhu et al. 2016 [26].
6	*IER3*	9104	cg06126421	−0.101	2.6E−04	Allione et al. 2015 [11]; Guida et al. 2015 [12]; Besingi and Johansson 2014 [14]; Dogan et al. 2014[15]; Elliott et al. 2014 [16]; Tsaprouni et al. 2014[18]; Shenker et al. 2013[19]; Zeilinger et al. 2013 [22]; Zhu et al. 2016 [26].
		9132	cg14753356^f^	−0.062	1.7E−05	Guida et al. 2015 [12]; Besingi and Johansson 2014 [14]; Zeilinger et al. 2013 [22]; Zhu et al. 2016 [26].
		9227	cg24859433	−0.037	0.003	Guida et al. 2015 [12]; Besingi and Johansson 2014 [14]; Dogan et al. 2014[15]; Tsaprouni et al. 2014[18]; Zeilinger et al. 2013 [22]; Zhu et al. 2016 [26].
		9233	cg15342087	−0.030	0.009	Guida et al. 2015 [12]; Besingi and Johansson 2014 [14]; Tsaprouni et al. 2014[18]; Zeilinger et al. 2013 [22]; Zhu et al. 2016 [26].
7	*GNA12*		cg18446336	−0.074	0.011	Guida et al. 2015 [12]; Zhu et al. 2016 [26].
	*MYO1G*		cg19089201	0.056	3.5E−05	Zeilinger et al. 2013 [22]; Zhu et al. 2016 [26].
			cg22132788	0.092	2.7E−06	Guida et al. 2015 [12]; Besingi and Johansson 2014 [14]; Elliott et al. 2014 [16]; Zeilinger et al. 2013 [22]; Philibert et al. 2012[23]; Philibert et al. 2013[21]; Zhu et al. 2016 [26].
			cg04180046	0.103	2.3E−05	Zeilinger et al. 2013 [22]; Zhu et al. 2016 [26].
			cg12803068	0.156	4.8E−06	Allione et al. 2015 [11]; Guida et al. 2015 [12]; Besingi and Johansson 2014 [14]; Elliott et al. 2014 [16]; Zeilinger et al. 2013 [22]; Philibert et al. 2012[23]; Philibert et al. 2013[21]; Zhu et al. 2016 [26].
	*CNTNAP2*		cg21322436	−0.026	0.016	Guida et al. 2015 [12]; Zeilinger et al. 2013 [22]; Zhu et al. 2016 [26].
			cg25949550	−0.026	5.2E−05	Guida et al. 2015 [12]; Besingi and Johansson 2014 [14]; Zeilinger et al. 2013 [22]; Zhu et al. 2016 [26].
8	*MYST3*		cg14316231	−0.029	0.007	Guida et al. 2015 [12]; Zhu et al. 2016 [26].
9	*SLC44A1*	−1580	cg01692968^f^	−0.038	0.004	Guida et al. 2015 [12]; Besingi and Johansson 2014 [14]; Zeilinger et al. 2013 [22]; Zhu et al. 2016 [26].
10	*ZMIZ1*		cg03450842^f^	−0.041	0.004	Guida et al. 2015 [12]; Besingi and Johansson 2014 [14].
11	*KCNQ1OT1*		cg01744331	−0.030	8.4E−04	Guida et al. 2015 [12]; Zeilinger et al. 2013 [22].
			cg07123182^f^	−0.031	1.1E−05	Guida et al. 2015 [12]; Besingi and Johansson 2014 [14]; Elliott et al. 2014 [16]; Zeilinger et al. 2013 [22]; Zhu et al. 2016 [26].
			cg16556677^f^	−0.051	6.7E−04	Guida et al. 2015 [12]; Zeilinger et al. 2013 [22]; Zhu et al. 2016 [26].
			cg26963277^f^	−0.043	6.2E−04	Guida et al. 2015 [12]; Besingi and Johansson 2014 [14]; Zeilinger et al. 2013 [22]; Zhu et al. 2016 [26].
	*LRP5*		cg21611682	−0.045	0.005	Guida et al. 2015 [12]; Besingi and Johansson 2014 [14]; Zeilinger et al. 2013 [22]; Tsaprouni et al. 2014[18]; Zhu et al. 2016 [26].
			cg10420527	−0.031	0.013	Guida et al. 2015 [12]; Zhu et al. 2016 [26].
			cg14624207^f^	−0.040	0.002	Guida et al. 2015 [12]; Zeilinger et al. 2013 [22]; Zhu et al. 2016 [26].
	*ARRB1*		cg01901332	−0.057	0.008	Guida et al. 2015 [12]; Besingi and Johansson 2014 [14]; Zeilinger et al. 2013 [22].
	*PRSS23*		cg23771366	−0.062	0.002	Guida et al. 2015 [12]; Elliott et al. 2014 [16]; Zeilinger et al. 2013 [22]; Zhu et al. 2016 [26].
12	*ETV6*		cg07986378^f^	−0.069	3.6E−04	Guida et al. 2015 [12]; Besingi and Johansson 2014 [14]; Zhu et al. 2016 [26].
14	*C14orf43*		cg01731783	−0.025	0.009	Guida et al. 2015 [12]; Dogan et al. 2014[15]; Elliott et al. 2014 [16]; Zeilinger et al. 2013 [22].
	*ITPK1*		cg05284742	−0.055	2.5E−05	Guida et al. 2015 [12]; Besingi and Johansson 2014 [14]; Zeilinger et al. 2013 [22]; Zhu et al. 2016 [26].
15	*SEMA7A*		cg00310412	−0.036	0.008	Guida et al. 2015 [12]; Besingi and Johansson 2014 [14]; Zeilinger et al. 2013 [22].
	*ANPEP*		cg23161492	−0.055	0.001	Guida et al. 2015 [12]; Besingi and Johansson 2014 [14]; Zeilinger et al. 2013 [22]; Zhu et al. 2016 [26].
16	*XYLT1*		cg16794579^f^	−0.039	0.004	Guida et al. 2015 [12]; Besingi and Johansson 2014 [14].
	*FBRS*	−4029	cg07069636	−0.023	0.006	Guida et al. 2015 [12]; Zhu et al. 2016 [26].
17	*LOC100130933*		cg07251887^f^	−0.070	2.4E−04	Guida et al. 2015 [12]; Zeilinger et al. 2013 [22].
19	*CIRBP*	−1591	cg00073090	−0.031	0.002	Guida et al. 2015 [12]; Zeilinger et al. 2013 [22]; Zhu et al. 2016 [26].
	*MOBKL2A*		cg15187398	−0.048	0.013	Guida et al. 2015 [12]; Zeilinger et al. 2013 [22]; Zhu et al. 2016 [26].
	*MIR23A*	3767	cg05339037	−0.025	0.008	Guida et al. 2015 [12]; Zhu et al. 2016 [26].
	*F2RL3*		cg03636183^f^	−0.128	2.0E−08	Allione et al. 2015 [11]; Guida et al. 2015 [12]; Besingi and Johansson 2014 [14]; Dogan et al. 2014[15]; Harlid et al. 2014[17]; Elliott et al. 2014 [16]; Tsaprouni et al. 2014[18]; Shenker et al. 2013[19]; Sun et al. 2013[20]; Zeilinger et al. 2013 [22]; Wan et al. 2012[24]; Breitling et al. 2011[25]; Zaghlool et al. 2015[13]; Zhu et al. 2016 [26].
	*PPP1R15A*		cg03707168	−0.034	0.009	Guida et al. 2015 [12]; Besingi and Johansson 2014 [14].
20	*ATP9A*		cg07339236	−0.039	1.0E−04	Guida et al. 2015 [12]; Zeilinger et al. 2013 [22]; Zhu et al. 2016 [26].
21	*NCRNA00114*		cg06595162^f^	−0.034	0.005	Guida et al. 2015 [12]; Zeilinger et al. 2013 [22].
22	*NCF4*		cg02532700	−0.049	0.003	Guida et al. 2015 [12]; Zeilinger et al. 2013 [22]; Zhu et al. 2016 [26].

^a^Chromosome

^b^Distance to transcription start site of the mapped gene (basepair, National Center for Biotechnology Information human reference genome assembly Build 37.3)

^c^Regression coefficient from statistical model

^d^Statistical significance from statistical model

^e^Articles reporting CpGs as smoking-associated differential methylation sites at genome-wide level

^f^Probe differentially methylated in both current and former smokers compared to never smokers in our epigenome-wide association study

^g^Correction for 18 tests at the look-up

## Discussion

This is the second EWAS for smoking exposure in an East Asian population and the first which links differential methylation changes in blood to large-scale differential transcriptome profiles in lung tissue at multiple loci. We discovered novel smoking-associated DMRs as well as DMPs and confirmed previous findings mostly from non-Asian populations. We identified nominally significant correlations in DNA methylation in relation to quantitative measures of smoking: urine cotinine levels, pack-years, and duration of smoking cessation. Differentially expressed genes in relation to smoking intensity in lung tissue support the potential utility of our findings as blood DNA methylation biomarkers for smoking exposure.

We discovered 108 significant DMPs and 87 significant DMRs in relation to current smoking. Fourteen loci were significant from both approaches; nine of which were novel: *CALML4*, *CCND1*, *FOXK2*, *LINC01019*, *NKX2-3*, *NT5C1A*, *PRDM8*, *SPAG17*, and *SYNGR1*. It has been reported that genetic variants in *CCND1* and smoking exposure are associated with gastric carcinogenesis [53], nasopharyngeal carcinoma [54], and lung cancer [55] and useful for lung cancer prediction [56]. *PRDM8* encodes a protein which belongs to a conserved family of histone methyltransferases regulating transcription negatively.

Of the 87 significant DMRs, in 32 all CpGs were of nominal (*p* < 0.05) statistical significance. On average, 78 % of CpGs in each identified DMR were nominally significant. Although a DMR does not need to include a genome-wide significant DMP in the region, 14 DMRs contained FDR-significant DMPs. In our analysis of differentially methylated regions, the most highly significant DMRs consist of either one or two highly significant DMPs or closely spaced neighboring CpGs of only nominal statistical significance in the region (Additional file 6: Table S4). Although it has been reported that two methods that we used to identify DMRs can correct for irregular spacing of probes across the genome [44, 45], we cannot conclude whether these are reflecting true differential methylation or false discovery driven by array-design.

Our EWAS identified 104 DMPs from the analysis of current smokers that were also seen in former smokers compared to never smokers; 93 of which were novel. The methylation differences in current and former smokers compared to never smokers were only slightly attenuated. The persistence of blood DNA methylation changes in former smokers, even after 7 to 40 years of smoking cessation, is notable. Our analysis of duration of smoking cessation in former smokers showed positive correlations at seven loci—*IFI16*, *CLASP1*, *KTELC1*, *SPEF2*, *ACOT13*, *BSPRY*, and *FAM82A2*—which has not been previously reported in EWASs. We also found a negative correlation at cg25799109 in *ARHGEF3*, a known smoking-associated CpG [12].

Although there are biomarkers of current smoking, including nicotine and its metabolite cotinine levels in urine, blood, or saliva, biomarkers reflecting past smoking have been lacking. Interestingly, we found that most of the signals for current smoking remained for past smoking. Recent studies suggest that methylation signals are promising biomarkers for both current and lifetime smoking [57] that are related to mortality [58]. Significant methylation alterations in former smokers compared to never smokers from our study can contribute to development of biomarkers for past smoking.

For urinary cotinine, we confirmed previous findings of differential methylation at *GNG12*, *GPR15*, *F2RL3* [27], and *AHRR* [21, 27] at nominal statistical significance (*p* < 0.05) and negative directions of association were also consistent. We also identified novel positive and negative correlations with methylation levels at *MTNR1A* and *FAM82A2*, respectively. Gene-environment interactions of variants in *MTNR1A* and smoking have been reported in relation to oral cancer [59]. In studies without cotinine measured, differential methylation at loci correlated with cotinine could serve as objective biomarkers to confirm the self-reported current level of smoking. For pack-years, we found correlations with DNA methylation at *NT5C1A*, *ZBTB9*, *HPX*, *CCND1*, and *RNF160* which were have not been reported in previous EWASs. Although cg19134728 in *JAKMIP3* was previously shown to be differentially methylated in smokers compared to non-smokers [15], its relationship with pack-years in current smokers was never studied.

To gain some biological insight into the differential methylation from our EWAS, we linked our genome-wide significant results to large-scale transcriptome profiles in lung tissues. We discovered differential gene expressions in relation to pack-years at 20 genes which were mapped from 17 DMPs and 8 DMRs. Our findings include six genes—*GPR15*, *AHRR*, *LPP*, *GNA12*, *CYB561*, and *SNED1*—known for their association with smoking in previous EWASs, but none of these has been identified in transcriptome analyses of pack-years in lung tissue. Only one previous EWAS included smoking-associated differential gene expression at *AHRR*; that study included lung tissue samples from five smokers and five non-smokers [19].

Our finding of enrichment of significant DMPs in CpG island shore (regions within 2000 bp within a CpG island) is consistent with previous findings of variable DNA methylation in the regions [60], suggesting methylation in shore regions is more susceptible to environmental factors including smoking.

Our replication look-up confirmed 70 DMPs in the same direction of methylation changes from previous EWASs at strict look-up level significance. Of these, 51 were replicated in one EWAS [26] from a Chinese population. Nineteen were never replicated in an East Asian population. We could not replicate the novel findings identified from the EWAS in Chinese [26].

We had only one female current smoker and six male never smokers. Because of this imbalance, our adjustment for gender may not eliminate potential bias in the smoking results. We identified one EWAS of gender using Illumina’s 450k array [13] in blood DNA (*n* = 123). In their supplementary table, they presented 274 gender-associated CpGs of genome-wide significance (*p* < 1.07E−07) located in autosomes. None of our 108 smoking DMPs (FDR < 0.05) were among those suggesting that our top findings do not reflect the gender imbalance.

In our EWAS, we used COPD status as a covariate. The disease status could be a confounding factor. For 108 FDR-significant DMPs related to current smoking, we checked the association between COPD status and DNA methylation under two statistical models. Model 1 included covariates of age, sex, height, and estimated cell-type compositions; model 2 contained additional covariates of smoking status and pack-years. None of our DMPs were statistically significantly associated with COPD under either model (FDR ≤0.05 after correcting for 108 tests). Sixteen CpGs were nominally related to COPD at uncorrected *p* < 0.05 (Additional file 9: Table S9).

There are limitations and strengths in this study. First, these data were cross-sectional which limits causal inference regarding resolution of effects with cessation of smoking. Second, we do not have a replication dataset from an independent Korean, or similar, population. Therefore, there is a chance of false positives among our novel findings. Third, the study population was drawn from a COPD cohort. Although we adjusted for the disease status in the regression models, the possibility of some type of selection bias could be raised. Fourth, we used blood DNA methylation to examine effects of smoking. The use of blood DNA methylation changes can be limited due to cell- and tissue-specific characteristics of methylation. However, our findings of differential methylation were adjusted for estimated cell-type proportions. We also confirmed differential transcriptome patterns in relation to pack-years in lung tissue at multiple loci.

Our study also has strengths. This is one of the few studies in Asian populations and the first in Koreans. We verified self-reported non-smoking status with urine cotinine values. Underreporting of smoking status in surveys occurs [61] and the nondifferential misclassification could distort association results. We also implemented two DMR approaches to provide significant DMRs in our EWAS. The methodologies for the discovery of DMRs have been developed and revised over several years, and it has been reported that the performance of DMRcate and comb-p were superior to those of others [44]. We were also able to examine whether genes with differential methylation in relation to smoking also showed differential transcription in relation to smoking in lung tissue, an important target for smoking related pathology.

## Conclusions

Our study in Koreans, we discovered novel smoking-associated DNA methylation changes in blood and also confirmed many previous findings mostly identified in Caucasians. Observed correlations between methylation levels and quantitative measures of smoking exposures support the utility of blood DNA methylation biomarkers for smoking intensity and history. Our evaluation of differential gene expression profiles of corresponding genes in lung tissues supports the potential functional importance of our methylation findings.

## Additional files

Additional file 1: Table S2. Relevant parameters for differential methylated region calling. (DOC 32 kb) Additional file 2: Figure S2. Regional visualization of the association between current smoking and DNA methylation in blood. (DOC 555 kb) Additional file 3: Table S1. Epigenome-wide association results of current smoking (compared to never smoking). (XLSX 30455 kb) Additional file 4: Table S3. CpGs differentially methylated in blood DNA in relation to current smoking compared to never smoking: 108 probes (FDR <0.05, ordered by chromosomal location). (DOC 166 kb) Additional file 5: Figure S1. Manhattan plot and quantile-quantile plot. (DOC 468 kb) Additional file 6: Table S4. Table S4 CpGs included in the top five differentially methylated regions from each analysis: DMRcate and comb-p (ordered by software and chromosomal location). (DOC 146 kb) Additional file 7: Table S5. CpGs differentially methylated in blood DNA in relation to current and former smoking compared to never smoking, 104 probes (FDR^*^ <0.05, ordered by chromosomal location). (DOC 177 kb) Additional file 8: Table S6. Enriched networks in genes related to current smoking. (DOC 35 kb) Additional file 9: Table S9. Association results of COPD status and DNA methylation at the 16 CpGs with unadjusted *p* < 0.05 in Model 1 or 2. (DOC 47 kb) Additional file 10: Table S7. Top 30 CpGs differentially methylated in blood DNA in relation to current smoking compared to never smoking (FDR ≤ 0.05, ordered by *p* values). (DOC 69 kb) Additional file 11: Table S8. Differentially methylated regions in blood DNA in relation to current smoking compared to never smoking (multiple-testing corrected *p* < 0.01 at DMRcate and comb-p, ordered by *p* values). (DOC 173 kb)

## Abbreviations

*ACOT11*: Acyl-CoA thioesterase 11
*ACY3*: Aminoacylase 3
*ADAMTS2*: ADAM metallopeptidase with thrombospondin type 1 motif 2
*ADCYAP1R1*: ADCYAP receptor type I
*AHDC1*: AT-hook DNA binding motif containing 1
*AHRR*: Aryl-hydrocarbon receptor repressor
*ALDOA*: Aldolase, fructose-bisphosphate A
*ALOX15B*: Arachidonate 15-lipoxygenase, type B
*ALPPL2*: Alkaline phosphatase, placental-like 2
*BCL7C*: BCL tumor suppressor 7C
BMI: Body mass index
BMIQ: Beta MIxture Quantile dilation
bp: Basepair
*C11orf21*: Chromosome 11 open reading frame 21
*C20orf27*: Chromosome 20 open reading frame 27
C5orf63 (clone name: *FLJ44606*): Chromosome 5 open reading frame 63
*CALML4*: Calmodulin-like 4
*CASZ1*: Castor zinc finger 1
*CCDC57*: Coiled-coil domain containing 57
*CCND1*: Cyclin D1
*CD33*: CD33 molecule
*CD72*: CD72 molecule
*CDK2AP1*: Cyclin-dependent kinase 2 associated protein 1
CFAP36 (alias *CCDC104*): Cilia and flagella associated protein 36
*CFI*: Complement factor I
*CFL2*: Cofilin 2
*CIZ1*: CDKN1A interacting zinc finger protein 1
*CLASP1*: Cytoplasmic linker associated protein 1
COPD: Chronic obstructive pulmonary disease
*CORO2B*: Coronin 2B
CpG: Cytosine-phosphate-guanine
*CRISP2*: Cysteine rich secretory protein 2
*CYB561*: Cytochrome b561
*DDA1*: DET1 and DDB1 associated 1
*DEFA4*: Defensin alpha 4
*DGUOK*: Deoxyguanosine kinase
*DIXDC1*: DIX domain containing 1
DMP: Differentially methylated probe
DMR: Differentially methylated region
*E2F8*: E2F transcription factor 8
*EPB49*: Dematin actin binding protein
*EVL*: Enah/Vasp-like
EWAS: Epigenome-wide association study
*EXOC3L4*: Exocyst complex component 3-like 4
*F2RL3*: F2R-like thrombin/trypsin receptor 3
*FAM53B*: Family with sequence similarity 53 member B
FDR: False discovery rate
*FGFRL1*: Fibroblast growth factor receptor-like 1
*FOXK2*: Forkhead box K2
*GALNT2*: Polypeptide *N*-acetylgalactosaminyltransferase 2
GEO: Gene Expression Omnibus
*GFI1*: Growth factor independent 1 transcriptional repressor
*GLI4*: GLI family zinc finger 4
*GNA12*: G protein subunit alpha 12
*GNG12*: G protein subunit gamma 12
*GNG7*: G protein subunit gamma 7
*GPR15*: G protein-coupled receptor 15
*GRK5*: G protein-coupled receptor kinase 5
*HLA-DPB1*: Major histocompatibility complex, class II, DP beta 1
*IER3*: Immediate early response 3
*IFFO1*: Intermediate filament family orphan 1
*INSIG1*: Insulin-induced gene 1
IQR: Interquartile range
*IRF7*: Interferon regulatory factor 7
*JAML* (alias *AMICA1*): Junction adhesion molecule-like
*KIAA0182*: Gse1 coiled-coil protein
*KIAA1549L* (alias *C11orf41*): KIAA1549-like
*KRBOX1*: KRAB box domain containing 1
*KRT7*: Keratin 7
*LAIR1*: Leukocyte-associated immunoglobulin-like receptor 1
*LDLRAD4* (alias *C18orf1*): Low density lipoprotein receptor class A domain containing 4
*LGI1*: Leucine-rich glioma inactivated 1
*LGMN*: Legumain
limma: Linear Models for Microarray data
*LINC01019*: Long intergenic non-protein coding RNA 1019
*LPCAT1*: Lysophosphatidylcholine acyltransferase 1
*LPP*: LIM domain containing preferred translocation partner in lipoma
*LY6G6E*: Lymphocyte antigen 6 complex, locus G6E
*MAN2B1*: Mannosidase alpha class 2B member 1
*MGP*: Matrix Gla protein
*MIR155HG*: MIR155 host gene
*MXRA8*: Matrix remodeling associated 8
*MYO1G*: Myosin IG
*NEAT1*: Nuclear paraspeckle assembly transcript 1 (non-protein coding)
*NHEDC1*: Solute carrier family 9 member B1
*NKX2-3*: NK2 homeobox 3
*NT5C1A*: 5′-nucleotidase, cytosolic IA
*NTN1*: Netrin 1
*ODF3B*: Outer dense fiber of sperm tails 3B
*PAX8*: Paired box 8
*PCGF3*: Polycomb group ring finger 3
*PLEKHA8*: Pleckstrin homology domain containing A8
*PRDM8*: PR/SET domain 8
*PRR25*: Proline-rich 25
qRT-PCR: Quantitative real-time reverse transcription polymerase chain reaction
*RIN3*: Ras and Rab interactor 3
*RP11-474D1.3*: Retinitis pigmentosa 11
*SATB2*: SATB homeobox 2
*SCCPDH*: Saccharopine dehydrogenase
SD: Standard deviation
*SHISA8*: Shisa family member 8
*SLC16A12*: Solute carrier family 16 member 12
*SLFN12L*: Schlafen family member 12-like
SMCO1 (alias *C3orf43*): Single-pass membrane protein with coiled-coil domains 1
*SNCG*: Synuclein gamma
*SNED1*: Sushi-, nidogen-, and EGF-like domains 1
*SOX30*: SRY-box 30
*SPAG17*: Sperm-associated antigen 17
*STX2*: Syntaxin 2
*SYNGAP1*: Synaptic Ras GTPase activating protein 1
*SYNGR1*: Synaptogyrin 1
*TBCD*: Tubulin folding cofactor D
*THBS2*: Thrombospondin 2
*TIAM2*: T cell lymphoma invasion and metastasis 2
*TLE3*: Transducin-like enhancer of split 3
*TRAPPC9*: Trafficking protein particle complex 9
*TRG-AS1*: T cell receptor gamma locus antisense RNA 1
*TSPAN13*: Tetraspanin 13
*UTRN*: Utrophin
*ZBTB38*: Zinc finger and BTB domain containing 38
*ZC3H12D*: Zinc finger CCCH-type containing 12D
*ZNF385A*: Zinc finger protein 385A
*ZNF697*: Zinc finger protein 697
