# Supplementary material for: DNA methylation and smoking in Korean adults: epigenome-wide association study
Source: Clin Epigenetics. 2016 Sep 22;8:103. doi: 10.1186/s13148-016-0266-6 (PMC5034618; doi:10.1186/s13148-016-0266-6)
Supplement: Additional file 1: Table S2. — Relevant parameters for differential methylated region calling. (DOC 32 kb) [file 13148_2016_266_MOESM1_ESM.doc]

**Additional file 1:**

**Table S2. Relevant parameters for differential methylated region** calling.

| Software | DMRcate [1] | Comb-p [2] |
| --- | --- | --- |
| Minimum distance | lambda=1000 | –dist 1000 |
| Minimum number of probes | min.cpg =2 | –region-filter-n 2 |
| P cutoff to initiate a regional significance calculation | pcutoff = 0.05 | –seed 0.05 |
| Software specific options | p.adjusted.method = “BH”  c=2 |  |

1. Peters, T.J., et al., *De novo identification of differentially methylated regions in the human genome.* Epigenetics Chromatin, 2015. **8**: p. 6.

2. Pedersen, B.S., et al., *Comb-p: software for combining, analyzing, grouping and correcting spatially correlated P-values.* Bioinformatics, 2012. **28**(22): p. 2986-8.
