# Supplementary material for: DNA methylation and smoking in Korean adults: epigenome-wide association study
Source: Clin Epigenetics. 2016 Sep 22;8:103. doi: 10.1186/s13148-016-0266-6 (PMC5034618; doi:10.1186/s13148-016-0266-6)
Supplement: Additional file 2: Figure S2. — Regional visualization of the association between current smoking and DNA methylation in blood. (DOC 555 kb) [file 13148_2016_266_MOESM2_ESM.doc]

**Additional file 2:**

**Figure S2. Regional visualization of the association between current smoking and DNA methylation in blood .**

1. **Differential methylation at 2q37.1 (near *ALPPL2*)**

**
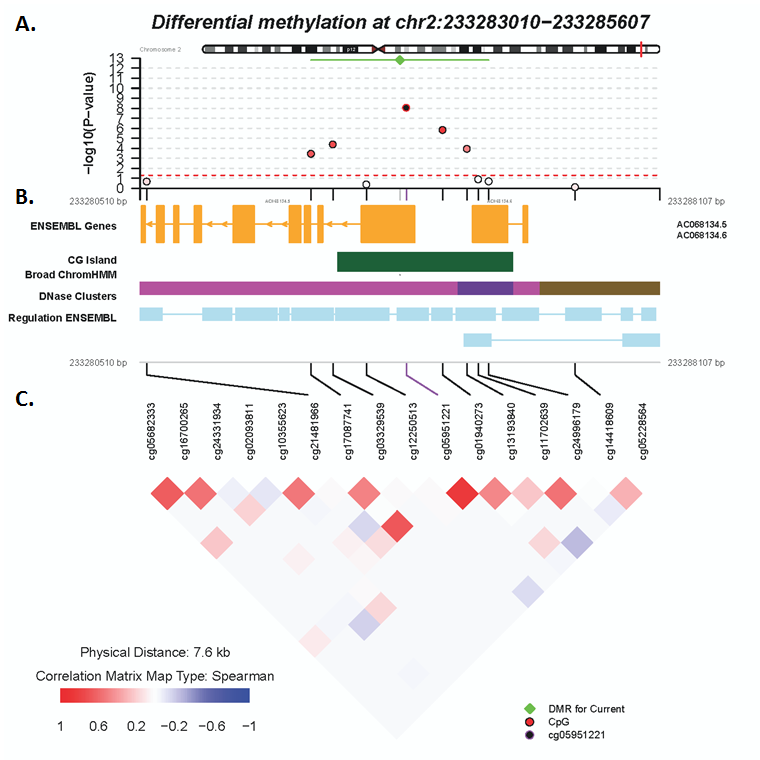
**

A: Regional association results of current versus never smoking. Statistical significance for the DMR in green represents FDR from DMRcate. CpGs are represented in circles with color indicating pairwise correlation with neighboring CpGs. Green horizontal bar with diamond shape represents differential methylation region of current smoking. B: Annotation tracks for the genomic region. Genes, regulatory region of the region from ENSEMBLE, Digital DNaseI Hypersensitivity Clusters from ENCODE (DNase Cluster), and Chromatin State Segmentation by HMM from ENCODE/Broad (Broad ChromHMM) are indicated. C: Pairwise correlation of CpGs in the region.

1. **Differential methylation in *PRDM8***

**
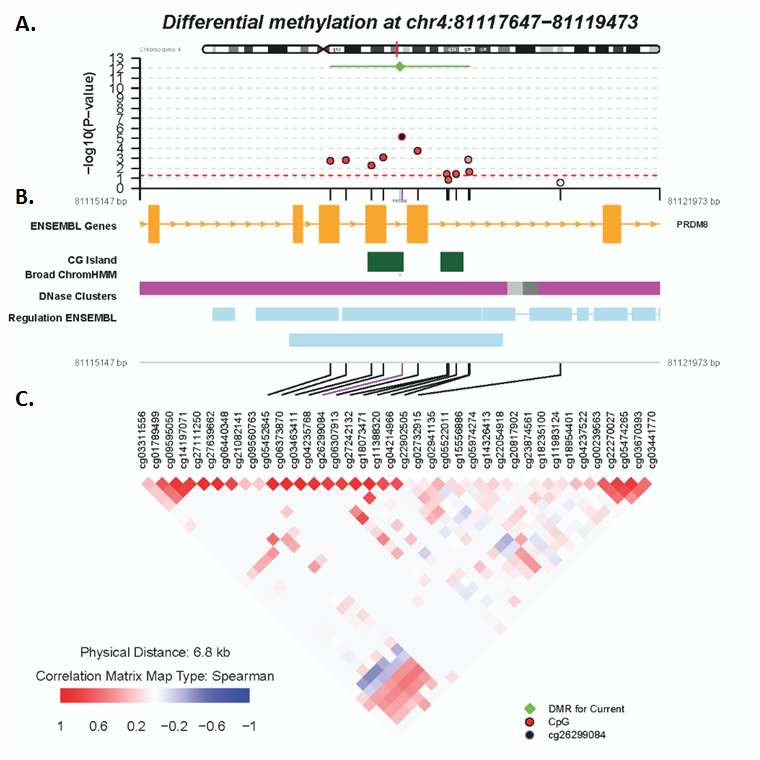
**

A: Regional association results of current versus never smoking. Statistical significance for the DMR in green represents FDR from DMRcate. CpGs are represented in circles with color indicating pairwise correlation with neighboring CpGs. Green horizontal bar with diamond shape represents differential methylation region of current smoking. B: Annotation tracks for the genomic region. Genes, regulatory region of the region from ENSEMBLE, Digital DNaseI Hypersensitivity Clusters from ENCODE (DNase Cluster), and Chromatin State Segmentation by HMM from ENCODE/Broad (Broad ChromHMM) are indicated. C: Pairwise correlation of CpGs in the region.

1. **Differential methylation in *NHEDC1***

**
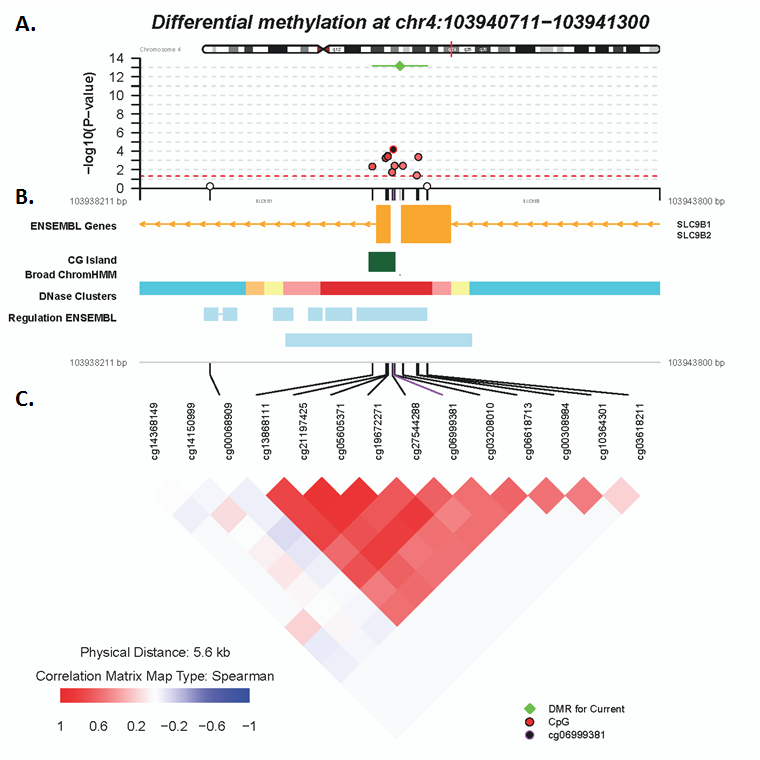
**

A: Regional association results of current versus never smoking. Statistical significance for the DMR in green represents FDR from DMRcate. CpGs are represented in circles with color indicating pairwise correlation with neighboring CpGs. Green horizontal bar with diamond shape represents differential methylation region of current smoking. B: Annotation tracks for the genomic region. Genes, regulatory region of the region from ENSEMBLE, Digital DNaseI Hypersensitivity Clusters from ENCODE (DNase Cluster), and Chromatin State Segmentation by HMM from ENCODE/Broad (Broad ChromHMM) are indicated. C: Pairwise correlation of CpGs in the region.

1. **Differential methylation in *ZC3H12D***

**
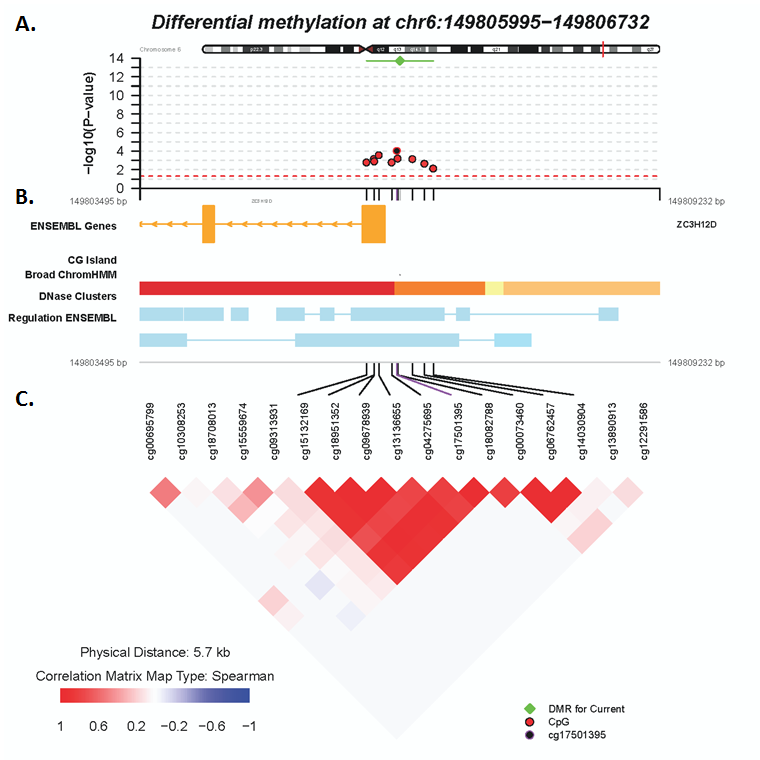
**

A: Regional association results of current versus never smoking. Statistical significance for the DMR in green represents FDR from DMRcate. CpGs are represented in circles with color indicating pairwise correlation with neighboring CpGs. Green horizontal bar with diamond shape represents differential methylation region of current smoking. B: Annotation tracks for the genomic region. Genes, regulatory region of the region from ENSEMBLE, Digital DNaseI Hypersensitivity Clusters from ENCODE (DNase Cluster), and Chromatin State Segmentation by HMM from ENCODE/Broad (Broad ChromHMM) are indicated. C: Pairwise correlation of CpGs in the region.

**Additional description of annotation tracks in Figure S2.**

Color coding in ‘Broad ChromHMM’ tracks are from UCSC genome browser (http://ucscbrowser.genap.ca/cgi-bin/hgTrackUi?db=hg19&g=wgEncodeBroadHmm).

State 1 - Bright Red - Active Promoter

State 2 - Light Red -Weak Promoter

State 3 - Purple - Inactive/poised Promoter

State 4 - Orange - Strong enhancer

State 5 - Orange - Strong enhancer

State 6 - Yellow - Weak/poised enhancer

State 7 - Yellow - Weak/poised enhancer

State 8 - Blue - Insulator

State 9 - Dark Green - Transcriptional transition

State 10 - Dark Green - Transcriptional elongation

State 11 - Light Green - Weak transcribed

State 12 - Gray - Polycomb-repressed

State 13 - Light Gray - Heterochromatin; low signal

State 14 - Light Gray - Repetitive/Copy Number Variation

State 15 - Light Gray - Repetitive/Copy Number Variation
