# Supplementary material for: DNA methylation and smoking in Korean adults: epigenome-wide association study
Source: Clin Epigenetics. 2016 Sep 22;8:103. doi: 10.1186/s13148-016-0266-6 (PMC5034618; doi:10.1186/s13148-016-0266-6)
Supplement: Additional file 5: Figure S1. — Manhattan plot and quantile-quantile plot. (DOC 468 kb) [file 13148_2016_266_MOESM5_ESM.doc]

**Additional file 5:**

**Figure S1. Manhattan plot and quantile-quantile plot.**

1. Manhattan plot of EWAS: current versus never smoking

**
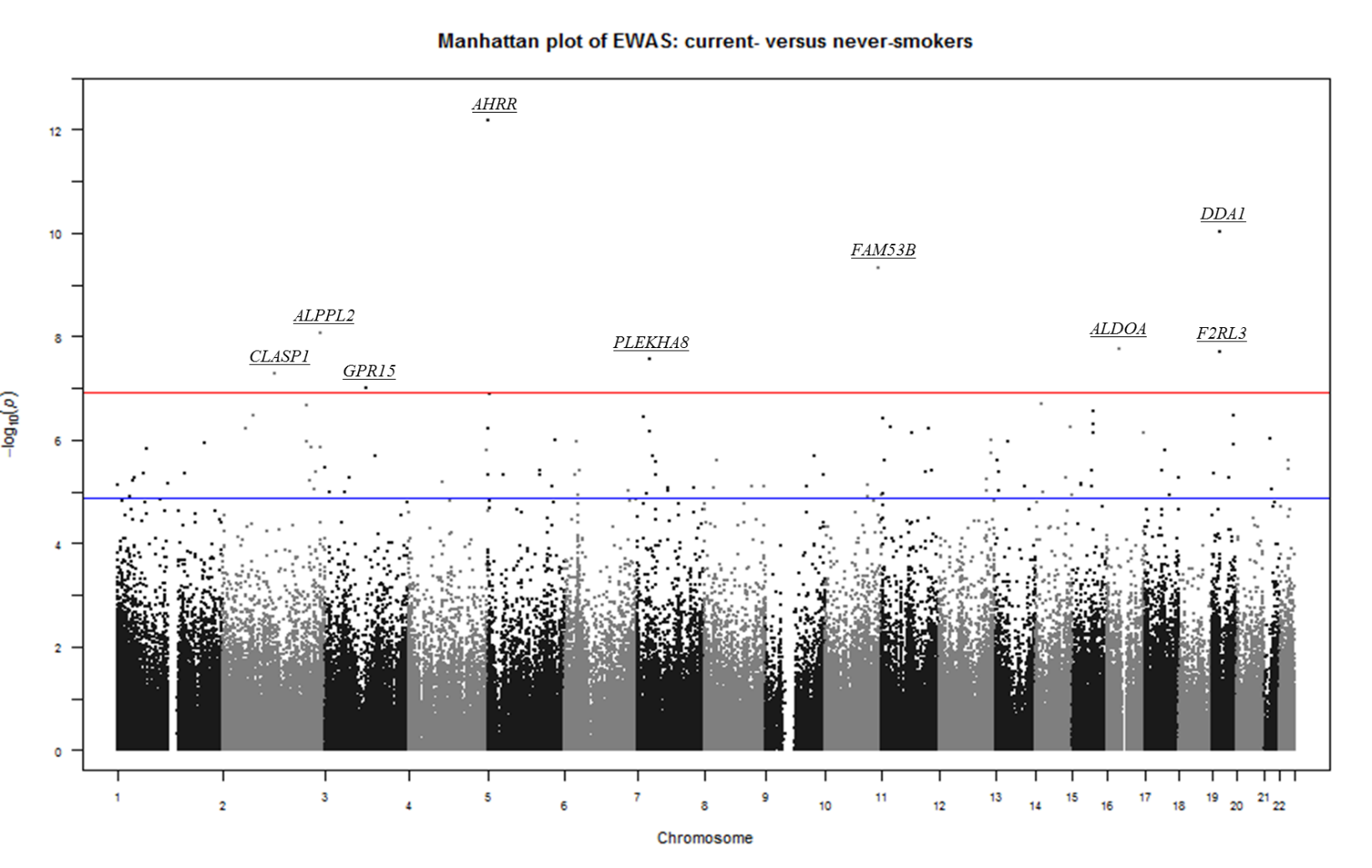
**

The manhattan plot shows –log_10_P on chromosomal locations. The horizontal lines in red and blue represent thresholds of genome-wide significance of Bonferroni (0.05/402508=1.2E-07) and false-discovery rate (FDR of 0.05), respectively. For CpGs of statistical significance after Bonferroni correction, gene names were added.

1. Quantile-quantile plot of EWAS: current versus never smoking

**
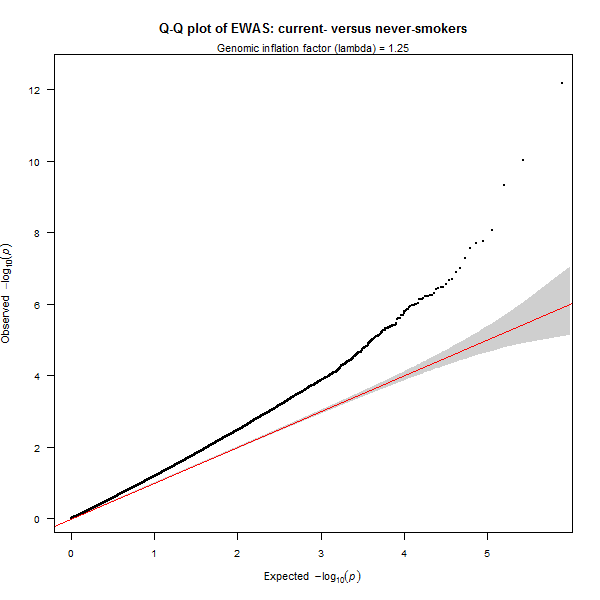
**

The Q-Q plot shows observed versus expected –log_10_P. Genomic inflation factor (lambda) was 1.25.
