# Supplementary material for: DNA methylation and smoking in Korean adults: epigenome-wide association study
Source: Clin Epigenetics. 2016 Sep 22;8:103. doi: 10.1186/s13148-016-0266-6 (PMC5034618; doi:10.1186/s13148-016-0266-6)
Supplement: Additional file 6: Table S4. — Table S4 CpGs included in the top five differentially methylated regions from each analysis: DMRcate and comb-p (ordered by software and chromosomal location). (DOC 146 kb) [file 13148_2016_266_MOESM6_ESM.doc]

**Additional file 6:**

**Table S4. CpGs included in the top five** differentially methylated regions from each analysis: DMRcate and comb-p (ordered by software and chromosomal location).

|  | Differentially methylated region | | | Differentially methylated probe | | | | | |
| --- | --- | --- | --- | --- | --- | --- | --- | --- | --- |
| Gene | Region^a^ | #CpGs^b^ | FDR or Sidak P^c^ | Probe | Position^d^ | P^e^ | Distance to gene^f^ | Gene feature^g^ | CpG island |
| DMRcate | | | | | | | | | |
| *ALPPL2* | 2:233283010-233285607 | 8 | 8.0E-15 | cg17087741 | 233283010 | 3.3E-04 | 11458 |  | shore |
|  |  |  |  | cg03329539 | 233283329 | 3.9E-05 | 11777 |  | shore |
|  |  |  |  | cg12250513 | 233283819 | 0.389 | 12267 |  | island |
|  |  |  |  | cg05951221 | 233284402 | 8.4E-09 | 12850 |  | island |
|  |  |  |  | cg01940273 | 233284934 | 1.4E-06 | 13382 |  | island |
|  |  |  |  | cg13193840 | 233285289 | 1.1E-04 | 13737 |  | island |
|  |  |  |  | cg11702639 | 233285454 | 0.119 | 13902 |  | island |
|  |  |  |  | cg24996179 | 233285607 | 0.191 | 14055 |  | island |
| *NHEDC1* | 4:103940711-103941300 | 11 | 6.8E-14 | cg13868111 | 103940711 | 0.004 |  | 5'UTR | island |
|  |  |  |  | cg21197425 | 103940854 | 5.2E-04 |  | 1stExon | island |
|  |  |  |  | cg05605371 | 103940876 | 3.0E-04 |  | TSS200 | island |
|  |  |  |  | cg19672271 | 103940878 | 3.6E-04 |  | TSS200 | island |
|  |  |  |  | cg27544288 | 103940923 | 0.017 |  | TSS200 | island |
|  |  |  |  | cg06999381 | 103940936 | 6.2E-05 |  | TSS200 | island |
|  |  |  |  | cg03208010 | 103940950 | 0.004 |  | TSS200 | island |
|  |  |  |  | cg06618713 | 103941039 | 0.004 |  | TSS200 | shore |
|  |  |  |  | cg00308984 | 103941189 | 0.038 |  | TSS1500 | shore |
|  |  |  |  | cg10364301 | 103941205 | 4.1E-04 |  | TSS1500 | shore |
|  |  |  |  | cg03618211 | 103941300 | 0.564 |  | TSS1500 | shore |
| *AHRR* | 5:373378-374425 | 5 | 4.6E-17 | cg05575921 | 373378 | 6.5E-13 |  | Body | shore |
|  |  |  |  | cg22103736 | 373887 | 0.315 |  | Body | island |
|  |  |  |  | cg08714121 | 374093 | 0.079 |  | Body | island |
|  |  |  |  | cg04141806 | 374252 | 6.6E-03 |  | Body | island |
|  |  |  |  | cg22356527 | 374425 | 0.995 |  | Body | island |
| *ZC3H12D* | 6:149805995-149806732 | 10 | 2.3E-15 | cg15132169 | 149805995 | 0.002 |  | 5'UTR |  |
|  |  |  |  | cg18951352 | 149806077 | 6.4E-04 |  | 5'UTR |  |
|  |  |  |  | cg09678939 | 149806081 | 0.001 |  | 5'UTR |  |
|  |  |  |  | cg13136655 | 149806131 | 2.6E-04 |  | 5'UTR |  |
|  |  |  |  | cg04275695 | 149806273 | 0.002 |  | TSS200 |  |
|  |  |  |  | cg17501395 | 149806331 | 8.7E-05 |  | TSS200 |  |
|  |  |  |  | cg18082788 | 149806339 | 6.0E-04 |  | TSS200 |  |
|  |  |  |  | cg00073460 | 149806502 | 6.7E-04 |  | TSS1500 |  |
|  |  |  |  | cg06762457 | 149806635 | 0.002 |  | TSS1500 |  |
|  |  |  |  | cg14030904 | 149806732 | 0.007 |  | TSS1500 |  |
| *MYO1G* | 7:45001765-45002919 | 6 | 5.5E-14 | cg03537938 | 45001765 | 0.033 | -495 |  | shore |
|  |  |  |  | cg02607319 | 45002112 | 0.929 | -148 |  | island |
|  |  |  |  | cg19089201 | 45002287 | 3.5E-05 |  | 3'UTR | island |
|  |  |  |  | cg22132788 | 45002486 | 2.7E-06 |  | Body | island |
|  |  |  |  | cg04180046 | 45002736 | 2.3E-05 |  | Body | island |
|  |  |  |  | cg12803068 | 45002919 | 4.8E-06 |  | Body | shore |
| Comb-p | | | | | | | | | |
| *ALPPL2* | 2:233283010-233285607 | 8 | 1.5E-13 | cg17087741 | 233283010 | 3.3E-04 | 11458 |  | shore |
|  |  |  |  | cg03329539 | 233283329 | 3.9E-05 | 11777 |  | shore |
|  |  |  |  | cg12250513 | 233283819 | 0.389 | 12267 |  | island |
|  |  |  |  | cg05951221 | 233284402 | 8.4E-09 | 12850 |  | island |
|  |  |  |  | cg01940273 | 233284934 | 1.4E-06 | 13382 |  | island |
|  |  |  |  | cg13193840 | 233285289 | 1.1E-04 | 13737 |  | island |
|  |  |  |  | cg11702639 | 233285454 | 0.119 | 13902 |  | island |
|  |  |  |  | cg24996179 | 233285607 | 0.191 | 14055 |  | island |
| *PRDM8* | 4:81117647-81119473 | 11 | 2.9E-13 | cg05452645 | 81117647 | 0.002 |  | TSS1500 | shore |
|  |  |  |  | cg06373870 | 81117853 | 0.001 |  | TSS1500 | shore |
|  |  |  |  | cg03463411 | 81118188 | 0.005 |  | TSS1500 | island |
|  |  |  |  | cg04235768 | 81118343 | 7.7E-04 |  | TSS1500 | island |
|  |  |  |  | cg26299084 | 81118588 | 6.7E-06 |  | 5'UTR | island |
|  |  |  |  | cg06307913 | 81118794 | 1.7E-04 |  | 5'UTR | shore |
|  |  |  |  | cg27242132 | 81119178 | 0.035 |  | 5'UTR | island |
|  |  |  |  | cg18073471 | 81119198 | 0.131 |  | 5'UTR | island |
|  |  |  |  | cg11388320 | 81119299 | 0.035 |  | 5'UTR | island |
|  |  |  |  | cg04214966 | 81119460 | 0.001 |  | 5'UTR | shore |
|  |  |  |  | cg22902505 | 81119473 | 0.021 |  | 5'UTR | shore |
| *NHEDC1* | 4:103940711-103941300 | 11 | 2.7E-10 | cg13868111 | 103940711 | 0.004 |  | 5'UTR | island |
|  |  |  |  | cg21197425 | 103940854 | 5.2E-04 |  | 1stExon | island |
|  |  |  |  | cg05605371 | 103940876 | 3.0E-04 |  | TSS200 | island |
|  |  |  |  | cg19672271 | 103940878 | 3.6E-04 |  | TSS200 | island |
|  |  |  |  | cg27544288 | 103940923 | 0.017 |  | TSS200 | island |
|  |  |  |  | cg06999381 | 103940936 | 6.2E-05 |  | TSS200 | island |
|  |  |  |  | cg03208010 | 103940950 | 0.004 |  | TSS200 | island |
|  |  |  |  | cg06618713 | 103941039 | 0.004 |  | TSS200 | shore |
|  |  |  |  | cg00308984 | 103941189 | 0.038 |  | TSS1500 | shore |
|  |  |  |  | cg10364301 | 103941205 | 4.1E-04 |  | TSS1500 | shore |
|  |  |  |  | cg03618211 | 103941300 | 0.564 |  | TSS1500 | shore |
| *ZC3H12D* | 6:149805995-149806732 | 10 | 1.9E-14 | cg15132169 | 149805995 | 0.002 |  | 5'UTR |  |
|  |  |  |  | cg18951352 | 149806077 | 6.4E-04 |  | 5'UTR |  |
|  |  |  |  | cg09678939 | 149806081 | 0.001 |  | 5'UTR |  |
|  |  |  |  | cg13136655 | 149806131 | 2.6E-04 |  | 5'UTR |  |
|  |  |  |  | cg04275695 | 149806273 | 0.002 |  | TSS200 |  |
|  |  |  |  | cg17501395 | 149806331 | 8.7E-05 |  | TSS200 |  |
|  |  |  |  | cg18082788 | 149806339 | 6.0E-04 |  | TSS200 |  |
|  |  |  |  | cg00073460 | 149806502 | 6.7E-04 |  | TSS1500 |  |
|  |  |  |  | cg06762457 | 149806635 | 0.002 |  | TSS1500 |  |
|  |  |  |  | cg14030904 | 149806732 | 0.007 |  | TSS1500 |  |
| *MYO1G* | 7:45001765-45002919 | 6 | 5.7E-09 | cg03537938 | 45001765 | 0.033 | -495 |  | shore |
|  |  |  |  | cg02607319 | 45002112 | 0.929 | -148 |  | island |
|  |  |  |  | cg19089201 | 45002287 | 3.5E-05 |  | 3'UTR | island |
|  |  |  |  | cg22132788 | 45002486 | 2.7E-06 |  | Body | island |
|  |  |  |  | cg04180046 | 45002736 | 2.3E-05 |  | Body | island |
|  |  |  |  | cg12803068 | 45002919 | 4.8E-06 |  | Body | shore |

^a^Region (chromosome:start-end, basepair)

^b^Number of probes in the region.

^c^False discovery rate for DMRcate or Sidak p for comb-p.

^d^Physical position (basepair, National Center for Biotechnology Information human reference genome assembly Build 37.3).

^e^Statistical significance from statistical model.

^f^Distance to transcription start site of the mapped gene (basepair).

^g^Categories for gene features includes (1) Body, gene body; (2) 5'UTR, 5 prime untranslated region; (3) 3'UTR, 3 prime untranslated region; (4) TSS200, 200 basepair within transcription start site; and (5) TSS1500, 1500 basepair within transcription start site.
