# Supplementary material for: DNA methylation and smoking in Korean adults: epigenome-wide association study
Source: Clin Epigenetics. 2016 Sep 22;8:103. doi: 10.1186/s13148-016-0266-6 (PMC5034618; doi:10.1186/s13148-016-0266-6)
Supplement: Additional file 7: Table S5. — CpGs differentially methylated in blood DNA in relation to current and former smoking compared to never smoking, 104 probes (FDR* <0.05, ordered by chromosomal location). (DOC 177 kb) [file 13148_2016_266_MOESM7_ESM.doc]

**Additional file 7:**

**Table S5. CpGs differentially methylated in blood DNA in relation to current and former smoking compared to never smoking: 104 probes (FDR^*^**<0.05, ordered by chromosomal location).

|  |  |  |  |  | Current vs. never smokers | | Former vs. never smokers | | |
| --- | --- | --- | --- | --- | --- | --- | --- | --- | --- |
| Chr^a^ | Gene | Distance to gene^b^ | Probe | Position^c^ | Coef^d^ | P^e^ | Coef | P | FDR^f^ |
| 1 | *HES4* |  | cg26321643 | 936914 | -0.013 | 7.4E-06 | -0.011 | 3.1E-04 | 5.0E-04 |
|  | *YTHDF2* |  | cg20388635 | 29063076 | -0.013 | 1.3E-05 | -0.011 | 1.9E-04 | 3.3E-04 |
|  | *MAP7D1* |  | cg00955686 | 36620450 | -0.016 | 6.3E-06 | -0.011 | 0.002 | 0.002 |
|  | *NT5C1A* |  | cg00990022 | 40138052 | -0.040 | 5.5E-06 | -0.041 | 7.1E-06 | 3.2E-05 |
|  | *NFIA* | -3590 | cg03223189 | 61539356 | -0.024 | 4.3E-06 | -0.021 | 9.6E-05 | 1.9E-04 |
|  | *GNG12* |  | cg25189904^g^ | 68299493 | -0.134 | 1.4E-06 | -0.082 | 0.003 | 0.004 |
|  | *SPAG17* |  | cg03850057 | 118727977 | -0.028 | 7.1E-06 | -0.027 | 2.7E-05 | 8.1E-05 |
|  | *IFI16* | -9970 | cg19707735 | 158969712 | -0.041 | 4.4E-06 | -0.035 | 1.0E-04 | 1.9E-04 |
|  | *AVPR1B* |  | cg08709672^g^ | 206224334 | -0.058 | 1.1E-06 | -0.030 | 0.012 | 0.013 |
| 2 | *CCDC104* |  | cg21597209 | 55746709 | -0.009 | 6.2E-07 | -0.006 | 0.002 | 0.003 |
|  | *DGUOK* |  | cg19394739 | 74154363 | -0.012 | 3.5E-07 | -0.009 | 2.3E-04 | 3.9E-04 |
|  | *CLASP1* |  | cg22346073 | 122402890 | -0.056 | 5.1E-08 | -0.052 | 8.0E-07 | 7.9E-06 |
|  | *SATB2* |  | cg21136715 | 200322252 | -0.035 | 2.1E-07 | -0.027 | 5.6E-05 | 1.3E-04 |
|  | *SPATS2L* |  | cg11912272 | 201204807 | -0.057 | 1.1E-06 | -0.048 | 6.9E-05 | 1.5E-04 |
|  | *ZDBF2* | -21270 | cg20471298 | 207118253 | -0.035 | 6.0E-06 | -0.027 | 6.3E-04 | 9.5E-04 |
|  | *LANCL1* |  | cg07063745 | 211341572 | -0.016 | 1.4E-06 | -0.013 | 8.5E-05 | 1.8E-04 |
|  | *WNT10A* |  | cg00821731 | 219744626 | -0.051 | 8.9E-06 | -0.047 | 6.9E-05 | 1.5E-04 |
|  | *DNPEP* | -15098 | cg09059267 | 220223082 | -0.099 | 4.2E-06 | -0.082 | 1.7E-04 | 3.0E-04 |
|  | *ALPPL2* | 12850 | cg05951221^g^ | 233284402 | -0.088 | 8.4E-09 | -0.053 | 2.9E-04 | 4.7E-04 |
|  |  | 13382 | cg01940273^g^ | 233284934 | -0.090 | 1.4E-06 | -0.039 | 0.034 | 0.036 |
| 3 | *CHL1* |  | cg04001014 | 238318 | -0.032 | 3.5E-06 | -0.027 | 1.1E-04 | 2.1E-04 |
|  | *IRAK2* | -3093 | cg10699312 | 10203470 | -0.080 | 1.0E-05 | -0.094 | 9.1E-07 | 8.2E-06 |
|  | *TREX1* |  | cg01870865 | 48507087 | -0.045 | 1.0E-05 | -0.033 | 0.001 | 0.002 |
|  | *ARHGEF3* |  | cg25799109^g^ | 57102900 | -0.084 | 5.3E-06 | -0.076 | 4.4E-05 | 1.1E-04 |
|  | *GPR15* |  | cg19859270^g^ | 98251294 | -0.027 | 1.0E-07 | -0.009 | 0.048 | 0.050 |
|  | *KTELC1* |  | cg16958524 | 119187511 | -0.030 | 2.1E-06 | -0.029 | 6.9E-06 | 3.2E-05 |
| 4 | *PRDM8* |  | cg26299084 | 81118588 | -0.095 | 6.7E-06 | -0.092 | 2.6E-05 | 8.0E-05 |
|  | *MTNR1A* |  | cg22261866 | 187475891 | -0.063 | 1.6E-06 | -0.041 | 0.002 | 0.002 |
| 5 | *AHRR* |  | cg05575921^g^ | 373378 | -0.203 | 6.5E-13 | -0.080 | 0.002 | 0.002 |
|  |  |  | cg14817490^g^ | 392920 | -0.078 | 4.7E-06 | -0.037 | 0.029 | 0.031 |
|  | *LINC01019* | -239389 | cg11405538 | 3177877 | 0.124 | 1.3E-07 | 0.107 | 6.9E-06 | 3.2E-05 |
|  | *SPEF2* |  | cg08534016 | 35771584 | -0.071 | 4.9E-06 | -0.050 | 0.001 | 0.002 |
|  | *LOX* |  | cg16274199 | 121414067 | 0.014 | 4.7E-06 | 0.009 | 0.002 | 0.002 |
|  | *CEP120* | -59650 | cg17807172 | 122620929 | -0.066 | 3.9E-06 | -0.056 | 1.4E-04 | 2.6E-04 |
|  | *PCYOX1L* |  | cg09838876 | 148737760 | 0.010 | 7.7E-06 | 0.008 | 3.8E-04 | 5.9E-04 |
|  | *SOX30* |  | cg06995810 | 157079468 | 0.048 | 1.0E-06 | 0.040 | 3.9E-05 | 1.0E-04 |
| 6 | *ACOT13* | 16438 | cg09447457 | 24721528 | -0.010 | 4.8E-06 | -0.010 | 1.2E-05 | 4.5E-05 |
|  | *LOC401242* | 15894 | cg09191776^h^ | 28843296 | 0.032 | 1.1E-06 | 0.032 | 2.8E-06 | 1.8E-05 |
|  | *NFKBIL1* |  | cg21053741 | 31525861 | -0.032 | 1.2E-05 | -0.031 | 3.4E-05 | 9.0E-05 |
|  | *ZBTB9* |  | cg03945003 | 33423747 | -0.023 | 3.9E-06 | -0.014 | 0.006 | 0.007 |
|  | *ESR1* |  | cg23164938 | 152128366 | -0.016 | 9.5E-06 | -0.017 | 1.3E-05 | 4.7E-05 |
| 7 | *TSPAN13* |  | cg05848863 | 16794078 | -0.024 | 3.6E-07 | -0.020 | 3.1E-05 | 8.6E-05 |
|  | *OSBPL3* |  | cg25270424 | 24965657 | 0.027 | 1.1E-05 | 0.029 | 4.8E-06 | 2.6E-05 |
|  | *PLEKHA8* |  | cg09762120 | 30108301 | 0.040 | 2.8E-08 | 0.044 | 3.7E-09 | 2.0E-07 |
|  | *ADCYAP1R1* |  | cg20165074 | 31091813 | -0.008 | 6.7E-07 | -0.007 | 3.2E-05 | 8.6E-05 |
|  | *ELMO1* |  | cg05383910^h^ | 37431792 | -0.042 | 2.1E-06 | -0.050 | 6.5E-08 | 1.0E-06 |
|  | *STX1A* |  | cg20663219 | 73130521 | -0.054 | 9.4E-06 | -0.056 | 1.1E-05 | 4.2E-05 |
|  | *LIMK1* |  | cg06126335 | 73497616 | -0.033 | 8.4E-06 | -0.031 | 2.8E-05 | 8.2E-05 |
|  | *BPGM* | 52093 | cg02821149 | 134383624 | -0.013 | 8.6E-06 | -0.012 | 7.8E-05 | 1.7E-04 |
| 8 | *NKX2-6* | 24440 | cg15820062 | 23584404 | -0.047 | 8.5E-06 | -0.045 | 2.9E-05 | 8.2E-05 |
|  | *DCTN6* |  | cg04374813 | 30012889 | 0.069 | 2.6E-06 | 0.037 | 0.011 | 0.012 |
|  | *CSMD3* |  | cg15430464 | 114450065 | -0.012 | 8.1E-06 | -0.008 | 0.003 | 0.003 |
|  | *SLC45A4* | 27388 | cg20657864^h^ | 142248258 | -0.055 | 7.9E-06 | -0.027 | 0.029 | 0.031 |
| 9 | *C9orf3* |  | cg14276379 | 97663142 | -0.113 | 7.8E-06 | -0.080 | 0.002 | 0.002 |
|  | *BSPRY* |  | cg02003202 | 116111459 | -0.051 | 2.1E-06 | -0.049 | 9.5E-06 | 3.8E-05 |
|  | *MIR4669* | -32167 | cg14321284 | 137186149 | -0.070 | 4.7E-06 | -0.073 | 4.3E-06 | 2.4E-05 |
| 10 | *NKX2-3* | -4844 | cg04972745 | 101287846 | -0.048 | 7.4E-06 | -0.055 | 1.3E-06 | 1.0E-05 |
|  |  |  | cg03609639 | 101291397 | -0.052 | 1.2E-05 | -0.052 | 2.0E-05 | 7.0E-05 |
|  | *FAM53B* |  | cg20723792 | 126360669 | -0.097 | 4.8E-10 | -0.130 | 1.2E-14 | 1.3E-12 |
| 11 | *IRF7* |  | cg27271532 | 612762 | -0.035 | 3.8E-07 | -0.024 | 3.5E-04 | 5.5E-04 |
|  | *KCNQ1OT1* |  | cg07123182^g^ | 2722391 | -0.031 | 1.1E-05 | -0.021 | 0.003 | 0.004 |
|  | *HPX* |  | cg25426350 | 6462391 | -0.030 | 2.5E-06 | -0.016 | 0.011 | 0.012 |
|  | *E2F8* |  | cg15604507 | 19263433 | -0.021 | 5.7E-07 | -0.021 | 2.2E-06 | 1.5E-05 |
|  | *CCND1* |  | cg09520904 | 69462943 | -0.036 | 7.5E-07 | -0.038 | 6.7E-07 | 7.2E-06 |
|  | *BIRC3* |  | cg14481222 | 102187974 | -0.014 | 4.1E-06 | -0.014 | 7.3E-06 | 3.2E-05 |
|  | *DIXDC1* |  | cg11471799 | 111807548 | -0.023 | 6.2E-07 | -0.016 | 6.7E-04 | 9.9E-04 |
|  | *PVRL1* |  | cg15741162 | 119587364 | 0.026 | 3.9E-06 | 0.024 | 2.5E-05 | 8.0E-05 |
| 12 | *FAM109A* |  | cg24530795 | 111807189 | -0.052 | 5.9E-06 | -0.055 | 3.1E-06 | 1.9E-05 |
|  | *TBX3* | 24830 | cg06530563 | 115132889 | -0.030 | 9.9E-06 | -0.029 | 2.4E-05 | 8.0E-05 |
|  | *CDK2AP1* |  | cg13421247 | 123756945 | -0.058 | 9.8E-07 | -0.059 | 2.1E-06 | 1.5E-05 |
|  | *TMEM132B* | -12436 | cg16901123 | 125798726 | -0.091 | 1.9E-06 | -0.071 | 2.4E-04 | 4.0E-04 |
| 13 | *CENPJ* |  | cg17058676 | 25468667 | -0.028 | 2.5E-06 | -0.023 | 9.2E-05 | 1.9E-04 |
|  | *GSX1* | -3499 | cg23019745 | 28363281 | 0.141 | 9.8E-06 | 0.098 | 0.002 | 0.003 |
|  | *POMP* | -37892 | cg16463452 | 29195249 | -0.067 | 4.3E-06 | -0.047 | 0.001 | 0.002 |
|  | *RB1* |  | cg08770358 | 48876684 | 0.016 | 1.1E-06 | 0.009 | 0.006 | 0.007 |
|  | *MIR622* | 117861 | cg18685745 | 91001297 | -0.033 | 7.9E-06 | -0.030 | 5.2E-05 | 1.2E-04 |
| 14 | *CFL2* | -44147 | cg23429457 | 35135441 | -0.040 | 2.0E-07 | -0.031 | 6.9E-05 | 1.5E-04 |
|  | *FOXA1* | 9833 | cg01087008 | 38068590 | -0.049 | 1.0E-05 | -0.042 | 1.5E-04 | 2.7E-04 |
|  | *GPR68* |  | cg05875421^g^ | 91709951 | -0.033 | 5.4E-06 | -0.015 | 0.032 | 0.034 |
|  | *EXOC3L4* | -20369 | cg04884342 | 103546112 | 0.020 | 5.6E-07 | 0.023 | 2.4E-08 | 7.9E-07 |
|  | *BRF1* |  | cg16579351 | 105708255 | -0.017 | 1.2E-05 | -0.010 | 0.008 | 0.009 |
| 15 | *FAM82A2* |  | cg21580007 | 41028734 | -0.067 | 7.3E-06 | -0.049 | 9.0E-04 | 0.001 |
|  |  |  | cg19440278 | 41047657 | 0.007 | 7.0E-06 | 0.006 | 2.1E-04 | 3.6E-04 |
|  | *KIAA0101* |  | cg03849685 | 64673798 | 0.007 | 8.0E-06 | 0.004 | 0.010 | 0.011 |
|  | *OAZ2* |  | cg14488391 | 64995133 | -0.014 | 4.0E-06 | -0.010 | 6.8E-04 | 9.9E-04 |
|  | *CALML4* |  | cg00388154 | 68498857 | -0.058 | 2.9E-07 | -0.048 | 2.6E-05 | 8.0E-05 |
|  | *CORO2B* |  | cg18765659 | 69018349 | -0.053 | 7.4E-07 | -0.042 | 1.0E-04 | 1.9E-04 |
|  | *TLE3* |  | cg06730438^h^ | 70355664 | -0.016 | 4.9E-07 | -0.013 | 4.8E-05 | 1.2E-04 |
| 16 | *ALDOA* |  | cg24780263 | 30064201 | -0.011 | 1.8E-08 | -0.010 | 4.9E-07 | 5.9E-06 |
|  | *KIAA0182* |  | cg26723054 | 85650522 | -0.038 | 7.2E-07 | -0.044 | 3.7E-08 | 7.9E-07 |
| 17 | *RARA* |  | cg00236832^h^ | 38465489 | -0.018 | 3.9E-06 | -0.020 | 1.2E-06 | 1.0E-05 |
|  | *HOXB9* | -1767 | cg25526913 | 46696752 | -0.008 | 1.6E-06 | -0.009 | 2.0E-07 | 2.7E-06 |
|  | *YPEL2* |  | cg13521620 | 57410687 | -0.052 | 1.2E-05 | -0.036 | 0.003 | 0.004 |
|  | *FOXK2* |  | cg07539709 | 80545454 | -0.076 | 5.5E-06 | -0.078 | 6.2E-06 | 3.2E-05 |
| 19 | *SAFB2* |  | cg11928000 | 5622738 | -0.011 | 4.6E-06 | -0.009 | 4.0E-04 | 6.1E-04 |
|  | *F2RL3* |  | cg03636183^g^ | 17000585 | -0.128 | 2.0E-08 | -0.063 | 0.004 | 0.005 |
|  | *DDA1* |  | cg10664184 | 17420304 | -0.028 | 9.2E-11 | -0.024 | 4.4E-08 | 7.9E-07 |
|  | *SAMD4B* |  | cg17384440 | 39832197 | -0.014 | 5.3E-06 | -0.009 | 0.004 | 0.005 |
|  | *CD33* |  | cg06861672 | 51727798 | -0.036 | 3.3E-07 | -0.040 | 4.1E-08 | 7.9E-07 |
|  | *PPP2R1A* |  | cg02339198 | 52693970 | -0.007 | 1.2E-06 | -0.006 | 9.2E-05 | 1.9E-04 |
| 21 | *MIR155HG* |  | cg03872783 | 26934885 | -0.008 | 9.7E-07 | -0.007 | 8.3E-06 | 3.4E-05 |
|  | *RNF160* |  | cg13662262 | 30364895 | -0.010 | 9.2E-06 | -0.008 | 3.4E-04 | 5.4E-04 |
| 22 | *SYNGR1* |  | cg14780837 | 39760267 | -0.050 | 2.5E-06 | -0.040 | 1.6E-04 | 2.9E-04 |
|  | *MGAT3* |  | cg26692811 | 39883347 | -0.023 | 3.7E-06 | -0.013 | 0.009 | 0.010 |

^a^Chromosome.

^b^Distance to transcription start site of the mapped gene (basepair).

^c^Physical position (basepair, National Center for Biotechnology Information human reference genome assembly Build 37.3).

^d^Regression coefficient from statistical model.

^e^Statistical significance from statistical model.

^f^False discovery rate.

^g^Probe identified in previous epigenome-wide association studies (EWASs) of smoking.

^h^Probe mapped to genes identified in previous EWASs of smoking.
