# Supplementary material for: DNA methylation and smoking in Korean adults: epigenome-wide association study
Source: Clin Epigenetics. 2016 Sep 22;8:103. doi: 10.1186/s13148-016-0266-6 (PMC5034618; doi:10.1186/s13148-016-0266-6)
Supplement: Additional file 8: Table S6. — Enriched networks in genes related to current smoking. (DOC 35 kb) [file 13148_2016_266_MOESM8_ESM.doc]

**Additional file 8:**

**Table S6. Enriched networks** in genes related to current smoking.

| Diseases and functions | Score^a^ | Focus molecules^b^ | Molecules in network |
| --- | --- | --- | --- |
| Gene Expression, Cellular Movement, Embryonic Development | 41 | 23 | ADNP, BCL11A, CENPJ, CEP120, CLASP1, Ctbp, CTBP2, E2F8, FOXA1, FOXK2, Hdac, HISTONE, HOXB9, IL-1R, IRAK, IRAK2, JARID2, MARCKS, MUC2, Mucin, N-cor, NFkB (complex), NFKBIL1, Pias, PRDM8, Rar, RARA, SATB2, SYT6, TBX3, TCF, TNIP1, TOLLIP, Ubiquitin, YTHDF2. |
| Cancer, Cellular Development, Organismal Injury and Abnormalities | 38 | 22 | BCL9, Cbp/p300, CCND1, CD3, CHL1, Ck2, Creb, DCUN1D1, EP400, estrogen receptor, Histone h3, Histone h4, Hsp90, IFI16, Interferon alpha, JMY, JUN, KIAA0101, LGALS3, MKI67, OSBPL3, PAG1, PFKP, PICALM, PRC1, RNA polymerase II, Rxr, SAFB2, Secretase gamma, SPATS2L, TCR, TERT, TLE3, TRRAP, TSC22D1. |
| Amino Acid Metabolism, Post-Translational Modification, Small Molecule Biochemistry | 36 | 21 | Ahr-aryl hydrocarbon-Arnt, AHRR, ALDOA, ATP1A3, CASZ1, Cdk, CDK2AP1, Cyclin D, Cyclin E, DDA1, DIXDC1, E2f, ERK1/2, GLUD1, GNG12, Hat, HCRT, HES4, histone deacetylase, HPX, LOX, LTN1, MGAT3, NECTIN1, NFIA, Nuclear factor 1, Rb, RNA polymerase iii, SAP30L, SWI-SNF, thymidine kinase, TRIB1, WLS, Wnt, WNT10A. |
| Hematological Disease, Metabolic Disease, Cardiovascular Disease | 34 | 20 | ADRB, Akt, ARHGEF3, BTNL2, CAMKK2, CDC14B, CFL2, CHRNA2, CHRNA7, Ciap, Cofilin, Collagen type I, GFPT1, Histone H1, MFNG, mir-101, nicotinic acetylcholine receptor, NKX2-3,NRG (family), NXN, PDGF BB, PI3K (family), PP1 protein complex group, PP2A, PPP1R2, Ppp2c, PPP2R1A, PRKAA, Rock, SCFD2, SIRT2, SLC25A11, TXNDC17, UNC5B, YPEL2. |
| Cell Signaling, Nucleic Acid Metabolism, Small Molecule Biochemistry | 27 | 17 | ADCYAP1R1, ADRB1, ARAP1, ARHGEF10, AVPR1B, Calmodulin, CORO2B, DEFA4, ELMO1, F Actin, F2RL3, G protein alphai, Gpcr, GPR15, GPR68, GPR152, GTPase, Hsp70, IFN alpha/beta, IL12 (family), LIMK1, MTNR1A, MTORC1, PI3K (complex), Pro-inflammatory Cytokine, Proinsulin, PTGER2, PTPRN2, Rac, Ras homolog, SGSM2, Sod, SRC (family), Tlr,tubulin (complex). |
| Reproductive System Development and Function, Developmental Disorder, Cancer | 21 | 14 | 26s Proteasome, ABLIM, Actin, AZIN2, BLCAP, C1QTNF5, CACNA1A, CALML4, Cg, DCTN6, EPHX4, ESR1, ETV2, FAM3D, Focal adhesion kinase, FSH, INSIG1, Insulin, LANCL1, Lh, LOC81691, Mapk, MOBP, OAZ2, ODC1-OAZ, OVOS2, P38 MAPK, Pka, RAD17, Ras, RB1, RBM12B, TOP1, TSPAN13, Vegf. |
| Lipid Metabolism, Molecular Transport, Small Molecule Biochemistry | 21 | 14 | APP, ART4, ATAT1, C11orf52, C14orf119, C2orf49, CSMD3, CYB561D2, DDX43, DGUOK, DTWD1, FAM109A, GTDC1, GUF1, HNF4A, METAP2, MTERF3, MYO1G, NEK4, omega-muricholic acid, PLEKHA8, PURG, RNASE7, RNH1, RSPH3, SLC35A3, SLC7A6OS, SNX21, SPAG17, SURF2, TBC1D12, TMTC4, TNF, TP53TG5, XPO1. |

^a^A score is derived from a p value in the Ingenuity Pathway Analysis software. The score of 20 represents a 1 in 1000 chance of having the Focus Molecules together in a network at random chance.

^b^Molecules are related to genes to which our significant differential methylation mapped among genes from each network in the Ingenuity Knowledge Base, Ingenuity Pathway Analysis (Ingenuity Systems, Redwood City, CA, USA, http://www.ingenuity.com/).
