# Supplementary material for: DNA methylation and smoking in Korean adults: epigenome-wide association study
Source: Clin Epigenetics. 2016 Sep 22;8:103. doi: 10.1186/s13148-016-0266-6 (PMC5034618; doi:10.1186/s13148-016-0266-6)
Supplement: Additional file 9: Table S9. — Association results of COPD status and DNA methylation at the 16 CpGs with unadjusted p < 0.05 in Model 1 or 2. (DOC 47 kb) [file 13148_2016_266_MOESM9_ESM.doc]

**Additional file 9. Table S9. Association results of COPD status and DNA methylation at** the 16 CpGs with unadjusted p<0.05 in Model 1 or 2

| Chr^a^ | Gene | Probe | EWAS of current smoking | Model 1 | Model 2 |
| --- | --- | --- | --- | --- | --- |
|  |  |  | P^b^ | P | P |
| 1 | *GNG12* | cg25189904 | 1.4E-06 | 0.03 | 0.07 |
| 1 | *IFI16* | cg19707735 | 4.4E-06 | 0.03 | 0.10 |
| 2 | *CCDC104* | cg21597209 | 6.2E-07 | 0.16 | 0.03 |
| 2 | *DGUOK* | cg19394739 | 3.5E-07 | 0.29 | 0.04 |
| 2 | *SATB2* | cg21136715 | 2.1E-07 | 0.25 | 0.03 |
| 2 | *LANCL1* | cg07063745 | 1.4E-06 | 0.12 | 0.01 |
| 2 | *DNPEP* | cg09059267 | 4.2E-06 | 0.07 | 0.02 |
| 3 | *ARHGEF3* | cg25799109 | 5.3E-06 | 0.01 | 0.04 |
| 6 | *ESR1* | cg23164938 | 9.5E-06 | 0.03 | 0.08 |
| 11 | *E2F8* | cg15604507 | 5.7E-07 | 0.20 | 0.04 |
| 13 | *CENPJ* | cg17058676 | 2.5E-06 | 0.09 | 0.01 |
| 14 | *CFL2* | cg23429457 | 2.0E-07 | 0.07 | 0.02 |
| 14 | *FOXA1* | cg01087008 | 1.0E-05 | 0.21 | 0.04 |
| 19 | *F2RL3* | cg03636183 | 2.0E-08 | 0.001 | 0.002 |
| 19 | *SAMD4B* | cg17384440 | 5.3E-06 | 0.07 | 0.02 |
| 21 | *MIR155HG* | cg03872783 | 9.7E-07 | 0.01 | 0.002 |

^a^Chromosome.

^b^Statistical significance from statistical model.

Model 1 included covariates of age, sex, height, and estimated cell-type compositions. Model2 contained additional covariates of smoking status and pack-years.
