# Supplementary material for: DNA methylation and smoking in Korean adults: epigenome-wide association study
Source: Clin Epigenetics. 2016 Sep 22;8:103. doi: 10.1186/s13148-016-0266-6 (PMC5034618; doi:10.1186/s13148-016-0266-6)
Supplement: Additional file 10: Table S7. — Top 30 CpGs differentially methylated in blood DNA in relation to current smoking compared to never smoking (FDR ≤ 0.05, ordered by p values). (DOC 69 kb) [file 13148_2016_266_MOESM10_ESM.doc]

**Additional file 10:**

Table S7. Top 30 CpGs differentially methylated in blood DNA in relation to current smoking compared to never smoking (FDR<0.05, ordered by p values)

| Chr^a^ | Gene | Distance to gene^b^ | Probe | Position^c^ | Coef^d^ | SE^e^ | P^f^ |
| --- | --- | --- | --- | --- | --- | --- | --- |
| 5 | *AHRR* |  | cg05575921^g^ | 373378 | -0.203 | 0.025 | 6.5E-13 |
| 19 | *DDA1* |  | cg10664184 | 17420304 | -0.028 | 0.004 | 9.2E-11 |
| 10 | *FAM53B* |  | cg20723792 | 126360669 | -0.097 | 0.014 | 4.8E-10 |
| 2 | *ALPPL2* | 12850 | cg05951221^g^ | 233284402 | -0.088 | 0.014 | 8.4E-09 |
| 16 | *ALDOA* |  | cg24780263 | 30064201 | -0.011 | 0.002 | 1.8E-08 |
| 19 | *F2RL3* |  | cg03636183^g^ | 17000585 | -0.128 | 0.021 | 2.0E-08 |
| 7 | *PLEKHA8* |  | cg09762120 | 30108301 | 0.04 | 0.007 | 2.8E-08 |
| 2 | *CLASP1* |  | cg22346073 | 122402890 | -0.056 | 0.01 | 5.1E-08 |
| 3 | *GPR15* |  | cg19859270^g^ | 98251294 | -0.027 | 0.005 | 1.0E-07 |
| 5 | *LINC01019* | -239389 | cg11405538 | 3177877 | 0.124 | 0.022 | 1.3E-07 |
| 14 | *CFL2* | -44147 | cg23429457 | 35135441 | -0.04 | 0.007 | 2.0E-07 |
| 2 | *SATB2* |  | cg21136715 | 200322252 | -0.035 | 0.006 | 2.1E-07 |
| 15 | *CALML4* |  | cg00388154 | 68498857 | -0.058 | 0.011 | 2.9E-07 |
| 19 | *CD33* |  | cg06861672 | 51727798 | -0.036 | 0.007 | 3.3E-07 |
| 2 | *DGUOK* |  | cg19394739 | 74154363 | -0.012 | 0.002 | 3.5E-07 |
| 7 | *TSPAN13* |  | cg05848863 | 16794078 | -0.024 | 0.004 | 3.6E-07 |
| 11 | *IRF7* |  | cg27271532 | 612762 | -0.035 | 0.006 | 3.8E-07 |
| 15 | *TLE3* |  | cg06730438^h^ | 70355664 | -0.016 | 0.003 | 4.9E-07 |
| 14 | *EXOC3L4* | -20369 | cg04884342 | 103546112 | 0.02 | 0.004 | 5.6E-07 |
| 11 | *E2F8* |  | cg15604507 | 19263433 | -0.021 | 0.004 | 5.7E-07 |
| 2 | *CCDC104* |  | cg21597209 | 55746709 | -0.009 | 0.002 | 6.2E-07 |
| 5 | *AHRR* |  | cg25648203^g^ | 395444 | -0.079 | 0.015 | 6.2E-07 |
| 11 | *DIXDC1* |  | cg11471799 | 111807548 | -0.023 | 0.004 | 6.2E-07 |
| 7 | *ADCYAP1R1* |  | cg20165074 | 31091813 | -0.008 | 0.002 | 6.7E-07 |
| 16 | *KIAA0182* |  | cg26723054 | 85650522 | -0.038 | 0.007 | 7.2E-07 |
| 15 | *CORO2B* |  | cg18765659 | 69018349 | -0.053 | 0.01 | 7.4E-07 |
| 11 | *CCND1* |  | cg09520904 | 69462943 | -0.036 | 0.007 | 7.5E-07 |
| 21 | *MIR155HG* |  | cg03872783 | 26934885 | -0.008 | 0.001 | 9.7E-07 |
| 12 | *CDK2AP1* |  | cg13421247 | 123756945 | -0.058 | 0.011 | 9.8E-07 |
| 5 | *SOX30* |  | cg06995810 | 157079468 | 0.048 | 0.009 | 1.0E-06 |

^a^Chromosome.

^b^Distance to transcription start site of the mapped gene (basepair).

^c^Physical position (basepair, National Center for Biotechnology Information human reference genome assembly Build 37.3).

^d^Regression coefficient from statistical model.

^e^Standard error of regression coefficient.

^f^Statistical significance from statistical model.

^g^Probe identified in previous epigenome-wide association studies (EWASs) of smoking.

^h^Probe mapped to genes identified in previous EWASs of smoking.
