# Supplementary material for: DNA methylation and smoking in Korean adults: epigenome-wide association study
Source: Clin Epigenetics. 2016 Sep 22;8:103. doi: 10.1186/s13148-016-0266-6 (PMC5034618; doi:10.1186/s13148-016-0266-6)
Supplement: Additional file 11: Table S8. — Differentially methylated regions in blood DNA in relation to current smoking compared to never smoking (multiple-testing corrected p < 0.01 at DMRcate and comb-p, ordered by p values). (DOC 173 kb) [file 13148_2016_266_MOESM11_ESM.doc]

**Additional file 11:**

**Table S8. Differentially methylated regions in blood DNA in relation to current smoking compared to never smoking (multiple-testing corrected p**<0.01 at DMRcate and comb-p, ordered by p values)

| Chr^a^ | Gene | Distance to gene^b^ | DMRcate | | | | Comb-p | | | | Minimum P^i^ |
| --- | --- | --- | --- | --- | --- | --- | --- | --- | --- | --- | --- |
|  |  |  | Start (bp^c^) | End (bp) | FDR^d^ | #CpGs^e^ | Start (bp) | End (bp) | Sidak p^f^ | #CpGs |  |
| 5 | *AHRR*^h^ |  | 373378^g^ | 374425 | 4.6E-17 | 5(2) |  | 373887 | 4.8E-05 | 2(1) | 6.5E-13 |
| 6 | *ZC3H12D*^h^ |  | 149805995 | 149806732 | 2.3E-15 | 10(10) |  |  | 1.9E-14 |  | 8.7E-05 |
| 2 | *ALPPL2*^h^ | 11458 | 233283010^g^ | 233285607 | 8.0E-15 | 8(5) |  |  | 1.5E-13 |  | 8.4E-09 |
| 7 | *MYO1G*^h^ |  | 45001765^g^ | 45002919 | 5.5E-14 | 6(5) |  |  | 5.7E-09 |  | 2.7E-06 |
| 4 | *NHEDC1* |  | 103940711 | 103941300 | 6.8E-14 | 11(10) |  |  | 2.7E-10 |  | 6.2E-05 |
| 4 | *PRDM8* |  | 81117647^g^ | 81119473 | 6.7E-13 | 11(10) |  |  | 2.9E-13 |  | 6.7E-06 |
| 11 | *ACY3* |  | 67418045 | 67418405 | 1.1E-09 | 12(11) |  |  | 8.7E-08 |  | 1.3E-04 |
| 6 | *HLA-DPB1* |  | 33047944 | 33049505 | 2.5E-09 | 20(15) |  |  | 4.8E-08 |  | 0.002 |
| 5 | *AHRR*^h^ |  | 392920^g^ | 393366 | 5.8E-08 | 3(3) |  |  | 3.9E-08 |  | 4.7E-06 |
| 17 | *FOXK2* |  | 80545020^g^ | 80545869 | 8.1E-08 | 11(6) |  |  | 2.6E-04 |  | 5.5E-06 |
| 22 | *SYNGR1* |  | 39759864^g^ | 39760267 | 1.2E-07 | 5(5) |  |  | 1.2E-06 |  | 2.5E-06 |
| 3 | *GPR15*^h^ |  | 98250723^g^ | 98251294 | 6.2E-07 | 2(1) | 98249859 |  | 6.2E-04 | 4(2) | 1.0E-07 |
| 1 | *GNG12*^h^ |  | 68298816^g^ | 68299511 | 7.0E-07 | 7(5) | 68299057 |  | 0.001 | 6(5) | 1.4E-06 |
| 5 | *FLJ44606* |  | 126408756 | 126409553 | 7.0E-07 | 13(11) |  |  | 1.9E-06 |  | 0.001 |
| 6 | *IER3*^h^ | 9104 | 30720080 | 30720491 | 1.2E-06 | 8(4) |  |  | 0.002 |  | 1.7E-05 |
| 2 | *SNED1*^h^ |  | 241975756 | 241976244 | 1.9E-06 | 4(4) |  |  | 3.8E-06 |  | 1.4E-04 |
| 7 | *TRG-AS1*^h^ | -29710 | 38350464 | 38351468 | 2.0E-06 | 7(6) |  |  | 1.1E-05 |  | 1.7E-04 |
| 11 | *CCND1* |  | 69462660^g^ | 69463323 | 2.4E-06 | 6(3) |  |  | 1.7E-04 |  | 7.5E-07 |
| 15 | *CALML4* |  | 68498251^g^ | 68499367 | 2.6E-06 | 5(2) | 68497992 |  | 0.002 | 6(2) | 2.9E-07 |
| 1 | *NT5C1A* |  | 40137636^g^ | 40138402 | 3.2E-06 | 6(3) |  |  | 0.001 |  | 5.5E-06 |
| 8 | *TRAPPC9* |  | 141057285 | 141057827 | 3.7E-06 | 5(5) |  |  | 2.1E-06 |  | 2.0E-04 |
| 7 | *GNA12*^h^ |  | 2768988 | 2770410 | 4.7E-06 | 5(5) | 2769253 |  | 7.4E-05 | 4(4) | 3.0E-05 |
| 6 | *CRISP2* |  | 49681178 | 49681774 | 5.5E-06 | 9(8) |  |  | 5.5E-06 |  | 1.8E-04 |
| 6 | *SYNGAP1*^h^ |  | 33400477 | 33401542 | 6.9E-06 | 9(7) | 33400021 |  | 2.2E-05 | 10(7) | 2.7E-04 |
| 10 | *NKX2-3* | -4844 | 101287381^g^ | 101287846 | 8.2E-06 | 5(3) |  |  | 1.3E-04 |  | 7.4E-06 |
| 11 | *C11orf21*^h^ |  | 2321770 | 2322674 | 1.2E-05 | 18(7) |  | 2323938 | 1.3E-04 | 33(8) | 5.9E-04 |
| 3 | *LPP*^h^ |  | 187870621 | 187871538 | 1.5E-05 | 11(5) |  |  | 0.001 |  | 1.1E-04 |
| 14 | *RIN3* |  | 92981121 | 92981666 | 1.6E-05 | 3(3) |  |  | 2.1E-05 |  | 1.8E-04 |
| 6 | *TIAM2*^h^ |  | 155537595 | 155538155 | 1.6E-05 | 8(5) |  |  | 3.7E-05 |  | 7.6E-04 |
| 17 | *NTN1* |  | 9018806 | 9019336 | 2.0E-05 | 5(4) |  |  | 5.5E-04 |  | 5.3E-04 |
| 11 | *NEAT1*^h^ | 4664 | 65194933 | 65196227 | 2.2E-05 | 7(7) |  | 65196696 | 3.0E-05 | 10(7) | 4.9E-04 |
| 6 | *LY6G6E* |  | 31683051 | 31683352 | 5.4E-05 | 6(5) |  |  | 1.2E-04 |  | 0.002 |
| 14 | *EVL* |  | 100610071 | 100610667 | 9.8E-05 | 6(4) |  |  | 1.9E-04 |  | 4.0E-04 |
| 17 | *CCDC57* |  | 80076338 | 80076378 | 1.1E-04 | 2(2) |  |  | 0.002 |  | 2.2E-05 |
| 1 | *GFI1*^h^ |  | 92946700 | 92947961 | 1.1E-04 | 6(4) |  |  | 1.2E-04 |  | 8.1E-05 |
| 17 | *ALOX15B* |  | 7942137 | 7942743 | 1.1E-04 | 6(5) |  |  | 2.4E-04 |  | 3.9E-04 |
| 1 | *SPAG17* |  | 118727658^g^ | 118728226 | 1.3E-04 | 10(2) |  |  | 0.005 |  | 7.1E-06 |
| 22 | *SHISA8* | -978 | 42304331 | 42304580 | 1.4E-04 | 2(2) |  |  | 6.9E-04 |  | 2.3E-05 |
| 4 | *FGFRL1* | -1776 | 1003208 | 1003834 | 1.5E-04 | 3(2) |  |  | 0.002 |  | 2.0E-04 |
| 10 | *SLC16A12*^h^ |  | 91296252 | 91296457 | 1.6E-04 | 3(3) |  |  | 0.004 |  | 4.4E-04 |
| 12 | *RP11-474D1.3* | 36620 | 130554977 | 130555091 | 1.8E-04 | 3(3) |  |  | 9.4E-04 |  | 1.7E-04 |
| 19 | *LAIR1* |  | 54876446 | 54876795 | 1.8E-04 | 5(4) |  |  | 8.1E-04 |  | 2.3E-04 |
| 8 | *DEFA4* |  | 6795162 | 6796618 | 2.0E-04 | 4(4) | 6794872 |  | 1.7E-05 | 5(4) | 4.0E-05 |
| 1 | *AHDC1* |  | 27929092 | 27929260 | 2.2E-04 | 2(2) |  |  | 0.006 |  | 1.5E-04 |
| 12 | *IFFO1* |  | 6657744 | 6658945 | 2.7E-04 | 10(5) |  | 6659524 | 2.2E-04 | 12(5) | 8.1E-05 |
| 22 | *ODF3B* |  | 50970943 | 50971140 | 4.2E-04 | 3(3) |  |  | 0.002 |  | 1.6E-04 |
| 17 | *CYB561* |  | 61511069 | 61511829 | 4.9E-04 | 4(4) |  |  | 9.3E-05 |  | 5.2E-04 |
| 10 | *SNCG* |  | 88717926 | 88718393 | 5.5E-04 | 5(5) |  |  | 3.8E-04 |  | 0.003 |
| 10 | *LGI1* |  | 95517382 | 95517895 | 6.3E-04 | 7(4) |  |  | 0.002 |  | 5.6E-04 |
| 10 | *GRK5* |  | 121171859 | 121172898 | 6.4E-04 | 5(4) |  |  | 2.4E-04 |  | 4.1E-04 |
| 11 | *C11orf41* |  | 33562503 | 33563377 | 7.0E-04 | 4(4) |  | 33563946 | 2.3E-04 | 5(4) | 5.4E-04 |
| 1 | *SCCPDH* | -26962 | 246859889 | 246860416 | 7.0E-04 | 5(4) |  |  | 2.6E-04 |  | 0.002 |
| 12 | *STX2* | -73033 | 131199848 | 131201112 | 7.2E-04 | 10(4) | 131198873 | 131201268 | 0.008 | 12(5) | 6.5E-05 |
| 1 | *CASZ1* | -600 | 10695686 | 10696066 | 8.7E-04 | 2(2) |  |  | 0.009 |  | 1.5E-05 |
| 12 | *MGP* |  | 15038440 | 15039432 | 9.5E-04 | 4(3) |  |  | 3.5E-05 |  | 9.3E-05 |
| 6 | *THBS2* |  | 169653612 | 169654719 | 9.5E-04 | 11(4) |  | 169654842 | 7.0E-04 | 12(4) | 5.3E-04 |
| 3 | *C3orf43* | 21882 | 196255632 | 196256223 | 9.7E-04 | 5(3) |  |  | 0.004 |  | 1.8E-04 |
| 3 | *KRBOX1* | 11 | 42977777 | 42978180 | 9.7E-04 | 7(5) |  |  | 0.003 |  | 4.1E-04 |
| 17 | *TBCD* |  | 80870107 | 80870923 | 0.001 | 5(3) |  | 80871405 | 0.002 | 7(4) | 1.8E-04 |
| 16 | *BCL7C* |  | 30906810 | 30907246 | 0.001 | 2(2) |  | 30907560 | 8.0E-04 | 3(3) | 9.0E-04 |
| 5 | *LPCAT1* |  | 1494980 | 1495356 | 0.001 | 5(4) |  |  | 0.003 |  | 0.001 |
| 9 | *CIZ1* |  | 130955135 | 130956057 | 0.001 | 4(3) |  | 130955436 | 0.004 | 3(3) | 0.001 |
| 8 | *GLI4* |  | 144358043 | 144359316 | 0.001 | 5(5) |  |  | 1.5E-05 |  | 0.002 |
| 4 | *PCGF3* |  | 737005 | 738199 | 0.002 | 8(2) | 736328 |  | 0.001 | 12(4) | 2.5E-05 |
| 14 | *LGMN* |  | 93170710 | 93170970 | 0.002 | 3(3) |  |  | 0.008 |  | 6.6E-05 |
| 20 | *C20orf27* |  | 3745817 | 3746315 | 0.002 | 2(2) |  |  | 0.004 |  | 8.8E-05 |
| 9 | *CD72* |  | 35609853 | 35610380 | 0.002 | 2(2) |  |  | 0.007 |  | 1.1E-04 |
| 12 | *KRT7* |  | 52638005 | 52638592 | 0.002 | 3(2) |  |  | 0.005 |  | 1.5E-04 |
| 1 | *MXRA8* | -812 | 1286917 | 1287259 | 0.002 | 2(2) |  |  | 0.002 |  | 2.2E-04 |
| 1 | *ACOT11*^h^ | -58441 | 54954187 | 54955366 | 0.002 | 7(4) | 54953632 |  | 0.009 | 8(4) | 6.1E-04 |
| 16 | *PRR25* |  | 854168 | 854640 | 0.002 | 4(3) |  | 855449 | 0.002 | 6(4) | 7.7E-04 |
| 5 | *ADAMTS2* |  | 178548229 | 178548700 | 0.002 | 3(3) |  |  | 0.003 |  | 8.5E-04 |
| 12 | *ZNF385A* |  | 54778312 | 54779175 | 0.002 | 4(3) |  |  | 0.008 |  | 0.001 |
| 1 | *GALNT2* |  | 230415343 | 230416101 | 0.002 | 6(3) | 230414987 | 230417096 | 1.2E-04 | 12(4) | 0.005 |
| 17 | *SLFN12L* | -13916 | 33787402 | 33788026 | 0.003 | 4(4) |  |  | 0.001 |  | 8.8E-04 |
| 6 | *UTRN* | -4373 | 144607399 | 144608500 | 0.004 | 7(4) | 144607074 |  | 0.01 | 8(4) | 2.6E-04 |
| 8 | *EPB49*^h^ |  | 21915184 | 21915510 | 0.004 | 2(2) | 21914287 | 21916853 | 5.3E-05 | 11(6) | 0.002 |
| 19 | *MAN2B1* |  | 12758416 | 12759546 | 0.004 | 7(4) |  |  | 0.001 |  | 0.002 |
| 11 | *AMICA1*^h^ |  | 118084920 | 118085736 | 0.005 | 4(4) |  |  | 0.002 |  | 0.003 |
| 2 | *PAX8* |  | 113992762 | 113993313 | 0.005 | 8(6) |  |  | 0.004 |  | 0.01 |
| 4 | *CFI* |  | 110724358 | 110724834 | 0.006 | 2(2) |  |  | 0.009 |  | 4.4E-04 |
| 5 | *LINC01019* | -236319 | 3180918 | 3180947 | 0.006 | 2(2) |  | 3182108 | 6.0E-04 | 5(4) | 5.7E-04 |
| 1 | *ZNF697* |  | 120173989 | 120174570 | 0.006 | 4(4) |  | 120174873 | 0.006 | 6(4) | 0.002 |
| 3 | *ZBTB38* |  | 141086820 | 141087363 | 0.006 | 6(4) |  |  | 0.005 |  | 0.005 |
| 7 | *INSIG1* | 61195 | 155150681 | 155151427 | 0.007 | 4(3) |  |  | 0.002 |  | 0.003 |
| 18 | *C18orf1* |  | 13611370 | 13611824 | 0.007 | 6(4) |  |  | 0.009 |  | 0.003 |
| 19 | *GNG7* |  | 2543602 | 2544100 | 0.008 | 5(2) | 2542837 |  | 0.002 | 6(3) | 6.4E-04 |

^a^Chromosome.

^b^Minimum distance to transcription start site of the mapped gene (basepair).

^c^Physical position (basepair, National Center for Biotechnology Information human reference genome assembly Build 37.3).

^d^False discovery rate.

^e^Number of probes in the region (Number of CpGs of nominal statistical significance).

^f^P of Sidak multiple-testing correction.

^g^Region including significant (FDR<0.05) differentially methylated probes from our epigenome-wide association study (EWAS).

^h^Gene identified in previous EWASs of smoking.

^i^Minimum p values among nominal p values of CpGs in each region.

Empty cells in ‘Start’, ‘End’, and ‘#CpGs’ for comb-p represent the same regional information compare to results in DMRcate.
